# HGFL-mediated RON signaling supports breast cancer stem cell phenotypes via activation of non-canonical β-catenin signaling

**DOI:** 10.18632/oncotarget.19441

**Published:** 2017-07-22

**Authors:** Sasha J. Ruiz-Torres, Nancy M. Benight, Rebekah A. Karns, Elyse E. Lower, Jun-Lin Guan, Susan E. Waltz

**Affiliations:** ^1^ Department of Cancer Biology, University of Cincinnati College of Medicine, Cincinnati, OH 45267, USA; ^2^ Division of Bioinformatics, Cincinnati Children's Hospital Medical Center, Cincinnati, OH 45229, USA; ^3^ Department of Internal Medicine, University of Cincinnati Medical Center, Cincinnati, OH 45267, USA; ^4^ Research Service, Cincinnati Veterans Affairs Medical Center, Cincinnati, OH 45267, USA

**Keywords:** RON receptor tyrosine kinase, HGFL, breast cancer stem cells, β-CATENIN, breast cancer

## Abstract

Breast cancer stem cells (BCSCs), which drive tumor progression, recurrence, and metastasis, are considered a major challenge for breast cancer treatments, thus the discovery of novel pathways regulating BCSC maintenance remains essential to develop new strategies to effectively target this population and combat disease mortality. The HGFL-RON signaling is overexpressed in human breast cancers and is associated with increased breast cancer progression, metastasis, and poor prognosis. Here, we report that overexpression of RON/MST1R and HGFL/MST1 in cell lines and primary tumors increases BCSC self-renewal, numbers, and tumorigenic potential after syngeneic transplantation. Transcriptome analyses also reveal that the HGFL-RON signaling pathway regulates additional BCSC functions and supports an immunosuppressive microenvironment to stimulate tumor formation and progression. Moreover, we show that genetic and chemical downregulation of HGFL-RON signaling disrupts BCSC phenotypes and tumor growth by suppressing the RON-mediated phosphorylation/activation of β-CATENIN/CTNNB1 and its effector NF-κB/RELA. These studies indicate that HGFL-RON signaling regulates BCSC phenotypes and the tumor microenvironment to drive tumorigenesis and present HGFL/RON as novel therapeutic targets to effectively eradicate BCSCs in patients.

## INTRODUCTION

Breast cancer is the most frequently diagnosed cancer and the second leading cause of cancer-related deaths among women in the United States, with 29% of women expected to develop invasive breast cancer and 14% of patients expected to die from this disease during 2016 [1]. Current treatments against breast cancer include surgery and use of conventional therapies (i.e. chemotherapy and targeted therapy) [1]. While advances in early detection and therapies have decreased breast cancer death rates, 20%–30% of patients develop tumor recurrence and therapeutic resistance, leading to advanced metastatic disease and significant mortality [2–5]. This highlights the lack of effectiveness of current treatments and the need to understand the underlying mechanisms mediating breast cancer progression to aggressive disease to develop effective treatments to combat disease mortality [6, 7].

Increasing evidence demonstrates that a subpopulation of cells within breast tumors, known as Breast Cancer Stem Cells (BCSCs) or breast cancer-initiating cells, drives breast cancer initiation and progression due to their increased self-renewal, survival, and metastatic potential as well as by generating the non-tumorigenic rapidly proliferating bulk cells [4, 8–11]. Additionally, BCSCs show resistance to conventional treatments since these therapies target the rapidly proliferating bulk cells, mediating therapeutic resistance, disease recurrence, and metastasis [4, 8, 10–14]. BCSCs are characterized by the ability to grow as self-renewing mammospheres under 3D-non-adherent conditions over several passages, expression of several cell surface markers (such as human Lin^−^CD44^+^CD24^−^ and murine Lin^−^CD29^Hi^CD24^+^), increased aldehyde dehydrogenase (ALDH) activity, and the ability to form tumors in hosts after transplantation into the mammary gland [8–15]. The majority of transplantation studies have been performed using xenografts of human BCSCs in immunocompromised mice [9, 11, 14]. However, several reports suggest that defects in the immune system and tumor microenvironment affect BCSC tumor initiation and growth, supporting the use of syngeneic models to better recapitulate the tumor microenvironment and BCSC niche [11, 14]. Despite extensive research, BCSCs remain a major obstacle in the clinic since current therapies have not effectively reduced BCSCs in patients [11, 13]. Therefore, the identification of novel molecular pathways supporting BCSC maintenance is critical to develop anti-cancer drugs targeting BCSCs to effectively combat breast cancer progression and improve the long-term survival of these patients [9, 11, 13, 14].

The RON receptor/MST1R is a member of the Met family of receptor tyrosine kinases and is primarily expressed in macrophages and epithelial cells [16]. Binding of its ligand, the Hepatocyte Growth Factor-Like protein (HGFL/MST1), to RON leads to RON activation and stimulates downstream signaling cascades, such as PI3K/AKT, MAPK, and β-CATENIN/CTNNB1, resulting in diverse cellular functions, including cell proliferation, survival, migration, invasion, angiogenesis, and therapeutic resistance [16–23]. As a result, RON is found highly expressed in several human cancers [16]. RON is minimally expressed in normal breast epithelium, but is overexpressed in more than 50% of human breast cancers and is associated with increased breast cancer progression, metastasis, and poor prognosis [16, 20, 23–26]. We previously demonstrated that RON and HGFL play important roles in breast development and tumorigenesis, with alterations in HGFL-RON signaling affecting terminal end bud formation in the developing mammary gland, which contains mammary stem cells, and promoting breast cancer progression and metastasis in transgenic models of breast cancer [6, 17, 18, 27]. However, it is unclear whether HGFL-RON signaling activates BCSC phenotypes to induce aggressive breast cancer.

In this report, we examined the role of HGFL-RON signaling in promoting breast cancer growth through regulation of the BCSC population. Our data using primary tumors from transgenic mice with modulations in RON or HGFL expression shows that overexpression of RON/HGFL increases BCSC self-renewal and numbers and is associated with increased tumor burden. The effects of HGFL-RON signaling on the BCSC population and tumorigenesis were also validated using cell lines with modulations in RON/HGFL expression. Moreover, our RNA-Seq analyses identified novel pathways through which HGFL-RON signaling may regulate BCSC functions and the tumor microenvironment (TME) to enhance tumor initiation and progression. Finally, we demonstrate that genetic downregulation and chemical inhibition of HGFL-RON signaling disrupts BCSC phenotypes by suppressing RON-mediated phosphorylation/activation of β-CATENIN and its downstream effector NF-κB. In summary, these studies indicate that HGFL-RON signaling regulates BCSC phenotypes to drive tumorigenesis and suggest HGFL/RON as potential therapeutic targets to eradicate BCSCs in patients.

## RESULTS

### HGFL and RON expression correlate with the proportion and function of BCSCs in spontaneous breast cancer models

**Figure 1:**
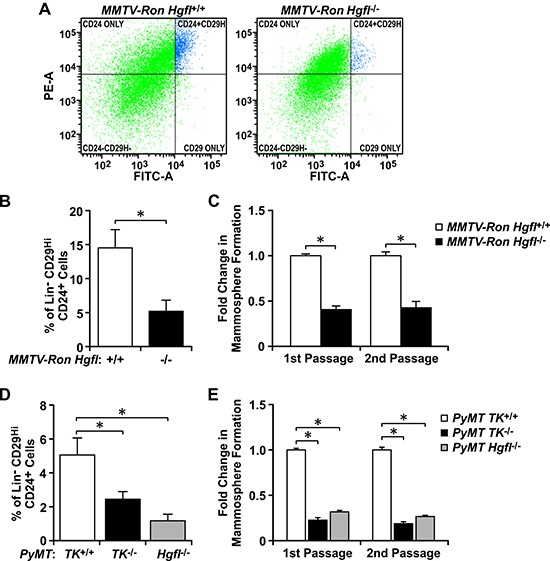
HGFL and RON expression correlate with the proportion and function of BCSCs in spontaneous breast cancer models (**A**) Representative images of the Lin^−^CD29^Hi^CD24^+^ flow cytometry analyses performed for *MMTV-Ron Hgfl*^+/+^ and *MMTV-Ron Hgfl*^−/−^ tumors. (**B**) Quantification of the percentage of Lin^−^CD29^Hi^CD24^+^ cells present in *MMTV-Ron Hgfl*^+/+^ and *MMTV-Ron Hgfl*^−/−^ tumors (*n* = 7-9). (**C**) Fold change in mammosphere formation over two passages obtained for Lin^−^CD29^Hi^CD24^+^ BCSCs from *MMTV-Ron Hgfl*^+/+^ and *MMTV-Ron Hgfl*^−/−^ tumors (*n* = 3-6). (**D**) Percentage of Lin^−^CD29^Hi^CD24^+^ cells obtained for *PyMT TK*^+/+^, *PyMT TK*^−/−^, and *PyMT Hgfl*^−/−^ tumors (*n* = 5–7). (**E**) Fold change in mammosphere formation during first and second passage of culture obtained for Lin^−^CD29^Hi^CD24^+^ BCSCs from *PyMT TK*^+/+^, *PyMT TK*^−/−^, and *PyMT Hgfl*^−/−^ tumors (*n* = 3-6). Bars represent average values ± SEM. *P < 0.05.

To examine the importance of HGFL-dependent RON signaling in BCSCs, we first investigated the *in vivo* significance of HGFL-RON signaling in promoting breast cancer growth through regulation of the BCSC population using two distinct and well established murine models of spontaneous breast cancer. First, we evaluated the size and self-renewal ability of the BCSC population in the *MMTV-Ron* mouse model of breast cancer with and without modulations in HGFL expression [6, 18]. Flow cytometry analyses of similar sized mammary tumors showed significantly fewer Lin^−^CD29^Hi^CD24^+^ BCSCs in the RON signaling-deficient *MMTV-Ron Hgfl*^−/−^ tumors compared with *MMTV-Ron Hgfl*^+/+^ controls (Figure 1A and 1B). To further our analysis, we plated equal numbers of Lin^−^CD29^Hi^CD24^+^ BCSCs isolated from these tumors under 3D-conditions over two passages to evaluate their mammosphere formation ability. Genetic loss of HGFL resulted in diminished BCSC self-renewal compared to HGFL replete controls (Figure 1C). This correlates with published studies demonstrating that ablation of HGFL-RON signaling delays mammary tumor initiation in this model [6]. Similar studies were performed using the *PyMT* model of breast cancer with modulations in HGFL or RON tyrosine kinase (TK) expression [17], with *PyMT TK*^−/−^ and *PyMT Hgfl*^−/−^ tumors having less Lin^−^CD29^Hi^CD24^+^ BCSCs and reduced self-renewal compared to controls (Figure 1D and 1E). The diminished BCSC populations correlate with decreased tumor growth following genetic ablation of HGFL-RON signaling in the *PyMT* model [17]. Taken together, our studies utilizing spontaneous breast cancer models demonstrate that genetic loss of RON and HGFL leads to a decrease in tumor burden which is associated with a reduction in BCSC numbers and their self-renewal ability, suggesting HGFL-RON signaling as an important regulator of the BCSC population.

### Loss of HGFL-RON signaling diminishes BCSC mammosphere formation and self-renewal

**Figure 2:**
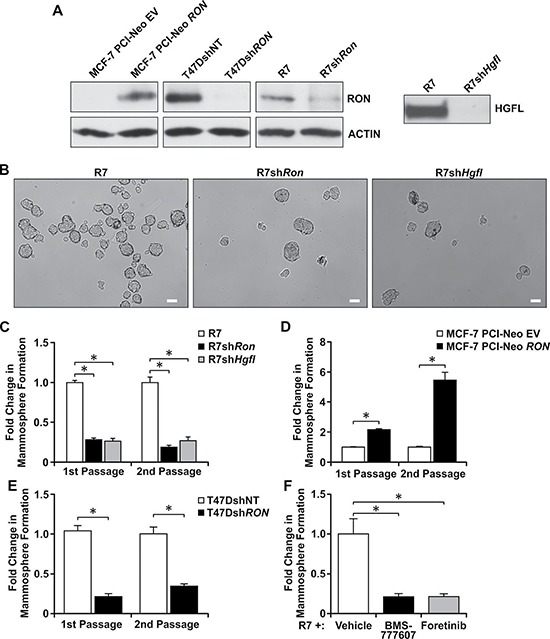
Loss of HGFL-RON signaling diminishes BCSC mammosphere formation and self-renewal (**A**) Left: Western blot images for RON and ACTIN expression in MCF-7, T47D, and R7 cells with modulations in RON expression. Right: Western analysis of conditioned media obtained from R7 control cells and cells with a knockdown of HGFL; HGFL is a secreted protein. (**B**) Representative images of mammospheres from R7, R7sh*Ron*, and R7sh*Hgfl* cells cultured under 3D-conditions. Scale bars=100μm. (**C**) Quantification of the change in mammosphere formation obtained for R7, R7sh*Ron*, and R7sh*Hgfl* cells cultured under 3D-conditions over two passages. (1st passage *n* = 3-5 and 2nd passage *n* = 2 independent experiments performed in triplicate). Mammosphere formation in the control cells was set to 1. (**D**) Fold change in mammosphere formation for MCF-7 PCI-Neo EV and MCF-7 PCI-Neo *RON* cells during first and second passage of suspension culture (*n* = 3-7 independent experiments performed in triplicate). (**E**) Fold change in mammosphere formation of T47Dsh*RON* cells compared to T47DshNT cells in passages 1 and 2 of culture (*n* = 3). (**F**) Fold change in mammosphere formation obtained for R7 cells after treatment with BMS-777607, Foretinib, or vehicle (*n* = 2 independent experiments performed in triplicate). Bars represent average values ± SEM. *P < 0.05.

We further investigated whether HGFL-RON signaling supports BCSC phenotypes using a panel of human and murine breast cancer cell lines with modulations in RON and HGFL expression. The efficiency of RON and HGFL modifications are demonstrated in Figure 2A. First, we tested the role of HGFL-RON signaling in regulating BCSC mammosphere formation and self-renewal by culturing the cells with modulations in RON/HGFL expression under 3D-non-adherent conditions over several passages. We initiated our studies utilizing a murine breast cancer cell line that expresses high levels of RON and HGFL (R7 cells) [6, 18, 19, 23]. No changes have been observed between parental R7 cells or R7 cells with a non-targeting (NT) control shRNA (Supplementary Figure 1A depicts no differences in cell growth between R7 cells either untransduced or transduced with a non-targeting (NT) control shRNA). In assessing the effect of HGFL and RON knockdown in R7 cells, we noticed that depletion of either protein resulted in markedly reduced mammosphere formation compared to HGFL and RON expressing control cells (Figure 2B and 2C). Knockdown of either protein resulted in a 3–4 fold decrease in sphere formation, which was observed over both first and second passages in culture (Figure 2C). This reduction in mammosphere formation was corroborated using a second stable RON knockdown cell line, R7 KD (3F7G10), obtained through CRISPR/CAS9 technology, with loss of RON resulting in a similar decrease in mammosphere formation (Supplementary Figure 1B and 1C). Interestingly, we also observed that addition of HGFL reverses the reduction in mammosphere formation ability of R7sh*Hgfl* cells. R7sh*Hgfl* cells treated with HGFL form a significantly higher number of mammospheres compared to R7sh*Hgfl*-vehicle treated cells (Supplementary Figure 1B), suggesting that stimulation of RON signaling rescues the BCSC self-renewal defect observed as a result of HGFL/RON loss.

Similar mammosphere formation studies were performed using human MCF-7 and T47D cells with modulations in RON expression (Figure 2A), with RON overexpressing MCF-7 PCI-Neo *RON* and T47DshNT cells forming a higher number of mammospheres compared to the RON depleted MCF-7 PCI-Neo EV and T47Dsh*Ron* cells (Figure 2D and 2E). These data suggest that BCSCs expressing high levels of RON and HGFL possess increased mammosphere formation and self-renewal abilities. We next tested the translational impact of inhibiting the HGFL-RON signaling pathway in BCSCs using two tyrosine kinase inhibitors with high selectivity for RON, namely BMS-777607 and Foretinib, which are currently in clinical trials. Both BMS-777607 and Foretinib efficiently block RON phosphorylation in R7 cells (Supplementary Figure 2A and 2B) [28–30]. When R7 mammospheres were treated with BMS-777607 or Foretinib, chemical inhibition of RON signaling significantly decreased the self-renewal ability of HGFL-RON expressing R7 BCSCs compared to vehicle-treated controls. Overall, these findings demonstrate that inhibition of HGFL-RON signaling is sufficient to reduce BCSC mammosphere formation and self-renewal abilities and strengthens the clinical significance of targeting this pathway in breast cancer patients.

### HGFL-RON signaling enhances BCSC numbers

**Figure 3:**
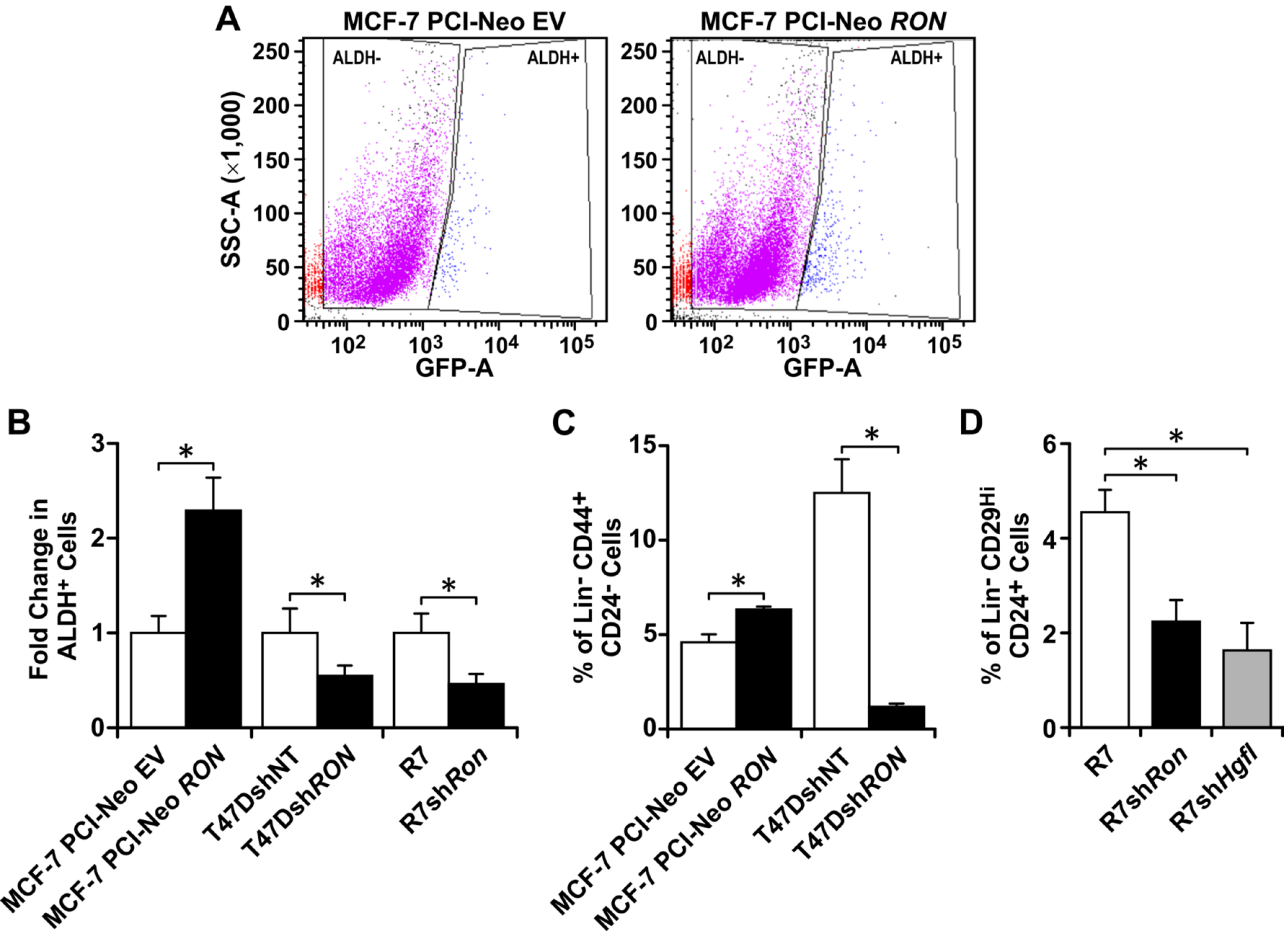
HGFL-RON signaling enhances BCSC numbers (**A**) Representative images of ALDH^+^ flow cytometry analyses performed on MCF-7 PCI-Neo EV and MCF-7 PCI-Neo *RON* cells subjected to the Aldefluor assay. (**B**) Quantification of the change in ALDH^+^ cells obtained for MCF-7 PCI-Neo EV, MCF-7 PCI-Neo *RON*, T47DshNT, T47Dsh*RON*, R7, and R7sh*Ron* cells (*n* = 3–6 per group). Each control cell line was normalized to 1. (**C**) Percentage of Lin^−^CD44^+^CD24^−^ cells obtained for the human MCF-7 PCI-Neo EV, MCF-7 PCI-Neo *RON*, T47DshNT, and T47Dsh*RON* cells (*n* = 3–4 per group). (**D**) Percentage of Lin^−^CD29^Hi^CD24^+^ cells obtained for the murine R7, R7sh*Ron*, and R7sh*Hgfl* cells (*n* = 3–6). Bars depict average values ± SEM. *P < 0.05.

Since BCSCs with high levels of RON and HGFL have enhanced self-renewal, we examined whether the expression of this signaling pathway also contributed to the growth of the BCSC population. This subpopulation of cancer cells can be isolated based on their Aldehyde Dehydrogenase (ALDH) enzymatic activity and the expression of different cell-surface markers, such as human Lin^−^CD44^+^CD24^−^ and murine Lin^−^CD29^Hi^CD24^+^ [9, 11, 13]. First, we evaluated the ALDH enzymatic activity of MCF-7 cells with modulations in RON expression by flow cytometry (Figure 3A). Quantification of ALDH^+^ cells is shown in Figure 3B, wherein a significant increase in ALDH^+^ cells was observed in MCF-7 cells with high RON expression compared to MCF-7 PCI-Neo EV control cells. Similar ALDH^+^ analyses were performed using T47D and R7 cells with modulations in RON expression. RON depleted T47Dsh*RON* and R7sh*Ron* cells exhibited fewer numbers of ALDH^+^ cells compared to RON expressing T47DshNT and R7 control cells (Figure 3B). To further support the data obtained by the Aldefluor assay, flow cytometry results were obtained utilizing the human Lin^−^CD44^+^CD24^−^ (Figure 3C) and the murine Lin^−^CD29^Hi^CD24^+^ BCSC markers (Figure 3D). RON or HGFL knockdown lead to decreased BCSC numbers based on marker expression, while ectopic RON expression in MCF-7 cells promoted BCSC numbers. Taken together, these studies indicate that HGFL-RON signaling promotes BCSC maintenance and enhances BCSC numbers.

### HGFL-RON signaling supports BCSC tumorigenic potential

An important BCSC property is their ability to form tumors following transplantation [7, 9, 11, 14]. As such, we performed limiting dilution transplantation assays (LDA) to examine whether HGFL-RON signaling regulates BCSC tumorigenic potential. A total of 10, 100, and 1000 Lin^−^CD29^Hi^CD24^+^ BCSCs sorted from R7, R7sh*Ron*, and R7sh*Hgfl* cells were injected into mammary fat pads of syngeneic mice and tumor formation was evaluated. While the three groups formed tumors in mice at a dilution of 1000 cells, the tumor formation of R7sh*Ron* and R7sh*Hgfl* BCSCs was significantly impaired at dilutions of 10 and 100 cells and the BCSC frequency was significantly reduced in these groups compared to R7 BCSC control (Table 1 and Supplementary Figure 3). This data suggests that loss of HGFL-RON signaling reduces the BCSC frequency and tumorigenic potential *in vivo*, consistent with the *in vitro* data demonstrating that R7 cells are enriched for BCSCs relative to R7sh*Ron* and R7sh*Hgfl* cells. Altogether, the data indicate that HGFL-RON signaling contributes to tumor initiation and growth by promoting BCSC self-renewal and tumor-initiating potential, consistent with our previous results (Figure 1).

**Table 1:** Tumor formation of HGFL-RON modulated Lin^–^CD29^Hi^CD24^+^ BCSCs following orthotopic transplantation into syngeneic FVB mice

BCSCs	Tumor Formation			BCSC frequency (95% Confidence Interval)
	10	100	1000	
R7	4/4	4/4	7/7	1/1 (1/15.6–1/1)
R7sh*Ron*	1/4	3/4	4/4	1/61.5 (1/184.2–1/20.5) *P* = 0.000436
R7sh*Hgfl*	1/4	3/4	4/4	1/61.5 (1/184.2–1/20.5) *P* = 0.000436

### HGFL-RON signaling in BCSCs alters genes involved in BCSC functions and in formulating the tumor microenvironment

Since our results demonstrate that HGFL-RON signaling regulates BCSC self-renewal and tumorigenic potential, we next investigated its potential role in regulating additional BCSC functions and the pathways involved in these regulations. For this, we identified a list of genes that are uniquely upregulated and downregulated in R7 Lin^−^CD29^Hi^CD24^+^ BCSCs compared to R7 parental cells and then examined how modulation in HGFL-RON signaling affected the expression of these genes. RNA-Seq analyses for R7 Lin^−^CD29^Hi^CD24^+^ BCSCs and R7 parental cells showed differential expression of 767 genes, with 474 genes being upregulated and 293 genes being downregulated in R7 Lin^−^CD29^Hi^CD24^+^ BCSCs (Supplementary Figure 4A). Ontology analyses revealed that R7 Lin^−^CD29^Hi^CD24^+^ BCSCs induce the transcription of developmental genes as well as of genes promoting BCSC functions (such as proliferation, migration, and adhesion), angiogenesis, extracellular matrix (ECM) organization, wound response, and the production of sterols and cytokines, whereas repress genes involved in steroid metabolism, natural killer cell proliferation, and T cell proliferation/differentiation compared to controls (Supplementary Figure 4B and 4C; Supplementary Tables 1 and 2), establishing the R7 Lin^−^CD29^Hi^CD24^+^ BCSCs as an aggressive tumor-initiating population that may modulate the TME to promote tumor growth.

**Figure 4:**
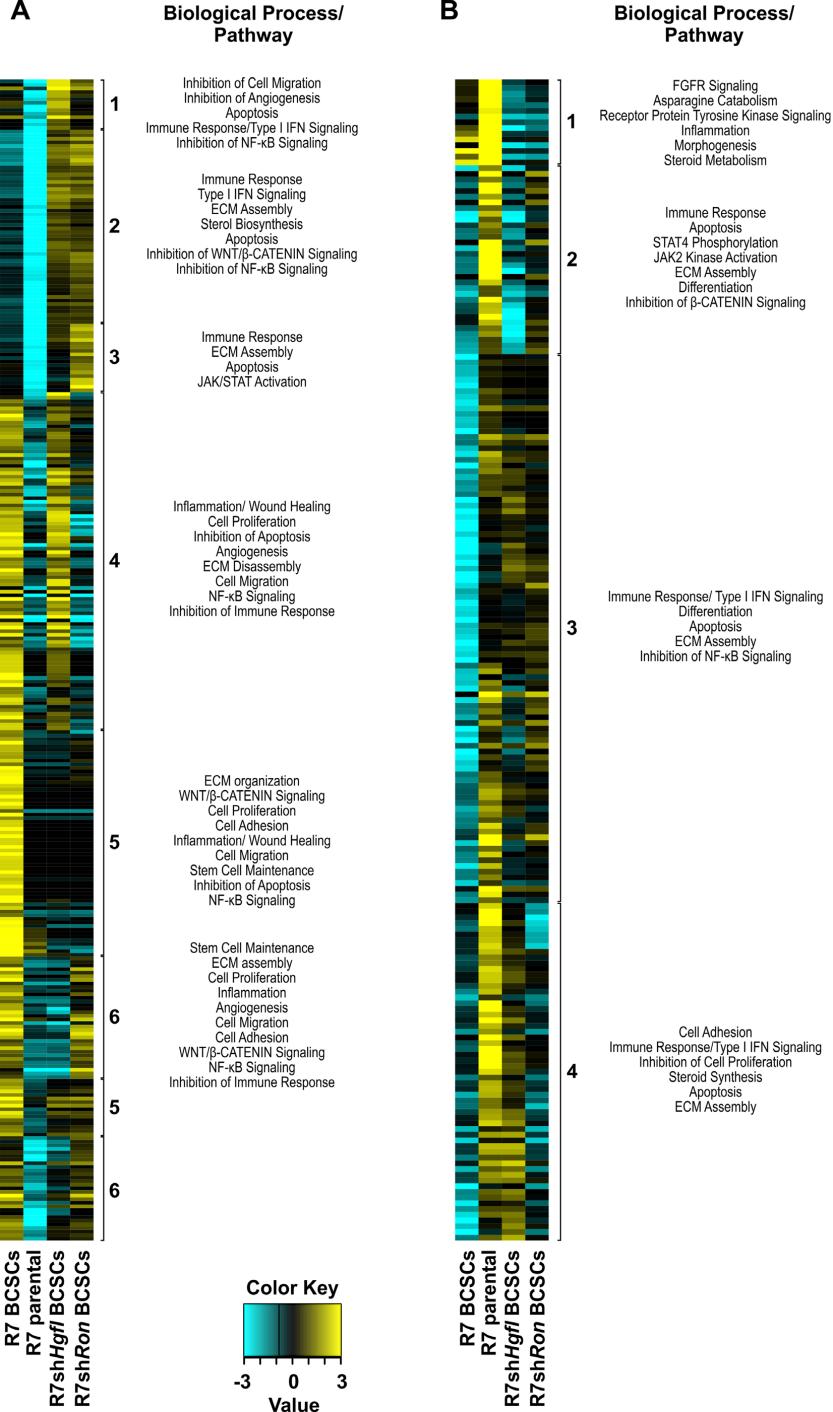
HGFL-RON signaling alters transcriptional programs in BCSCs that may impact BCSC phenotypes and the tumor microenvironment (**A**–**B**) Expression profile of uniquely upregulated (A) and downregulated BCSC genes (B) obtained for Lin^−^CD29^Hi^CD24^+^ BCSCs from R7, R7sh*Ron*, and R7sh*Hgfl* cells. Heatmaps depict normalized gene expression values for genes with > 2-fold change in expression in HGFL or RON deficient Lin^−^CD29^Hi^CD24^+^ BCSCs compared to R7 Lin^−^CD29^Hi^CD24^+^ BCSCs. R7 parental cells are shown for reference. Group clusters and the biological processes/pathways associated with each cluster are shown. See Supplementary Tables 3, 4, 5 for additional information.

To determine novel HGFL-RON signaling-dependent transcriptional networks that might regulate BCSC functions and the TME, we next examined RNA-Seq data for these differentially regulated 767 BCSC genes obtained for Lin^−^CD29^Hi^CD24^+^ BCSC populations from R7, R7sh*Ron*, and R7sh*Hgfl* cells (Figure 4A and 4B; Supplementary Tables 3, 4, 5). Ontology analyses demonstrated that loss of HGFL-RON signaling in BCSCs stimulates the transcription of genes mediating immune responses, cell differentiation, apoptosis, and ECM assembly, whereas suppresses BCSC functions (such as self-renewal/maintenance, proliferation, migration), angiogenesis, wound response, and sterol metabolism compared to R7 Lin^−^CD29^Hi^CD24^+^ BCSCs. Interestingly, we observed increased activation of anti-tumor immune-related pathways (STAT4/JAK2 and Type-I IFN signaling) and inhibition of BCSC regulators (β-CATENIN and NF-κB pathways) in HGFL-RON signaling-deficient BCSCs compared to controls. These data convincingly support the premise that HGFL-RON signaling stimulates BCSC functions through activation of β-CATENIN and NF-κB while supporting an immunosuppressive TME, resulting in increased BCSC maintenance and tumor progression.

### Loss of HGFL-RON activation in BCSCs correlates with decreased β-CATENIN and NF-κB signaling

**Figure 5:**
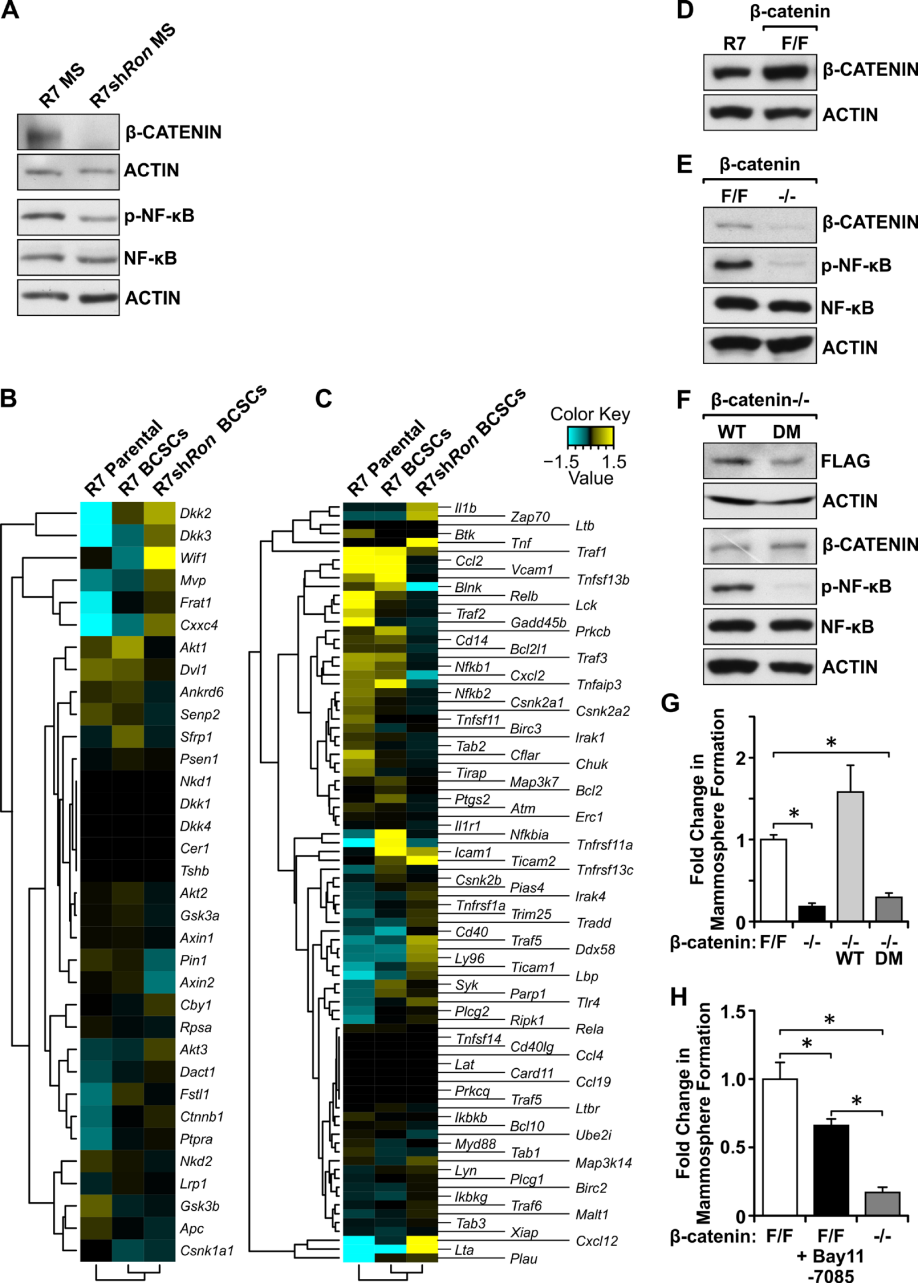
Loss of HGFL-RON signaling in BCSCs correlates with decreased β-CATENIN and NF-κB pathways (**A**) Western blot images for β-CATENIN, phosphorylated-NF-κB, NF-κB, and ACTIN protein levels in R7 and R7sh*Ron* BCSC-enriched mammospheres (MS). (**B**–**C**) Gene expression profile for β-CATENIN (B) and NF-κB (C) pathways in R7 Lin^−^CD29^Hi^CD24^+^ BCSCs, R7sh*Ron* Lin^−^CD29^Hi^CD24^+^ BCSCs, and R7 parental cells. See Supplementary Table 6 for additional information. (**D**) Western blot images showing β-CATENIN and ACTIN expression in R7 and β-cateninF/F mammary tumor cell lines. (**E**) Western blot images for β-CATENIN, phosphorylated-NF-κB, NF-κB, and ACTIN expression in β-cateninF/F and β-catenin−/− cells. (**F**) Western blot images showing FLAG, β-CATENIN, phosphorylated-NF-κB, NF-κB, and ACTIN expression in β-catenin−/− cells expressing exogenous WT and DM β-CATENIN. (**G**) Fold change in mammosphere formation obtained for β-cateninF/F, β-catenin−/−, β-catenin−/− WT, and β-catenin−/− DM cells cultured under 3D-conditions (*n* = 3-4). (**H**) Fold change in mammosphere formation for β-cateninF/F cells treated either with vehicle or 5μM Bay 11-7085 and β-catenin−/− cells (*n* = 2 independent experiments performed in triplicate). Bars depict average values ± SEM. *P < 0.05.

Next, we sought to elucidate the signaling differences between RON expressing and deficient BCSC-enriched mammospheres (MS) which are responsible for directing self-renewal and tumorigenic potential. Western analyses showed significant reductions in β-CATENIN and phosphorylated NF-κB in R7sh*Ron* BCSCs compared to controls (Figure 5A), suggesting these molecules are important regulators of BCSC phenotypes downstream of RON activation, consistent with our previous observations (Figure 4). To corroborate the involvement of these pathways in regulating BCSC phenotypes, we compared the β-CATENIN and NF-κB gene expression profiles between R7 parental, R7 Lin^−^CD29^Hi^CD24^+^ BCSCs, and R7sh*Ron* Lin^−^CD29^Hi^CD24^+^ BCSCs by RNA-Seq analysis (Figure 5B and 5C; Supplementary Table 6). Data mining revealed increased expression of β-CATENIN-associated genes (*Ctnnb1*, *Frat1*, and *Fstl1*) and a downregulation of β-CATENIN negative regulators (*Nkd2*, *Gsk3b*, and *Apc*) in the BCSC groups compared to R7 parental cells (Figure 5B and Supplementary Table 6), suggesting BCSCs have increased β-CATENIN signaling compared to parental cells. Comparing the Lin^−^CD29^Hi^CD24^+^ BCSC groups, we noticed a significant suppression of β-CATENIN target genes (*Akt1*/2, *Dvl1*, *Fstl1*, and *Pin1*) and an upregulation of β-CATENIN inhibitors (*Cby1*, *Wif1*, and *Dact1*) in R7sh*Ron* BCSCs compared to R7 BCSCs (Figure 5B and Supplementary Table 6), demonstrating higher β-CATENIN signaling in R7 BCSCs, consistent with our Western findings. Similar gene expression analyses performed for the NF-κB pathway demonstrated increased expression of NF-κB-associated genes (*Ccl2*, *Vcam1*, *Bcl2l1*, *Cxcl2*, and *Icam1*) and a downregulation of NF-κB anti-tumor genes (*Irak4*, *Tlr4*, *Ly96*, and *Lta*) in R7 BCSCs compared to R7sh*Ron* BCSCs (Figure 5C and Supplementary Table 6), validating that HGFL-RON signaling BCSCs have higher NF-κB signaling.

To define the requirement of β-CATENIN downstream of RON signaling, we employed breast cancer cells generated previously from a mammary tumor isolated from *MMTV-Ron* β*-catenin*^F/F^ mice, which contain floxed β-catenin alleles (β-cateninF/F) [23]. These cells express similar levels of β-CATENIN as the *MMTV-Ron*-derived R7 cells used in our previous studies (Figure 5D). To examine the role of HGFL-RON mediated β-CATENIN signaling in BCSCs, β-cateninF/F cells were infected with Adenovirus Cre-GFP to obtain cells deficient in β-CATENIN (β-catenin−/−) (Figure 5E) [23]. Surprisingly, we noted that loss of β-CATENIN resulted in a decrease in the phosphorylation of NF-κB (Figure 5E), suggesting β-CATENIN is regulating the activation of NF-κB downstream of RON to promote BCSC phenotypes. Mammosphere formation analyses using β-cateninF/F and β-catenin−/− cells showed that deficiency in β-CATENIN downstream of RON significantly diminishes BCSC self-renewal, with β-catenin−/− cells exhibiting ~80% reduction in mammosphere formation compared with controls (Figure 5G), validating β-CATENIN as a regulator of RON-mediated BCSC phenotypes. To further mechanistic analyses, we then examined whether NF-κB activation downstream of β-CATENIN is required to promote the RON-mediated BCSC phenotypes using the chemical inhibitor Bay 11-7085 [22]. Bay 11-7085 treatment of β-cateninF/F mammospheres efficiently blocked NF-κB phosphorylation without affecting the expression of RON or β-CATENIN (Supplementary Figure 5). Phenotypically, a significant decrease in mammosphere formation was observed after NF-κB inhibition, with β-cateninF/F Bay 11-7085-treated cells exhibiting ~35% reduction in mammosphere formation compared to vehicle-treated controls (Figure 5H). However, this inhibition was not sufficient to mimic the reduction in mammosphere formation obtained in β-catenin−/− cells (Figure 5H), suggesting that β-CATENIN promotes BCSC phenotypes upon RON activation partially through the activation of NF-κB.

**Figure 6:**
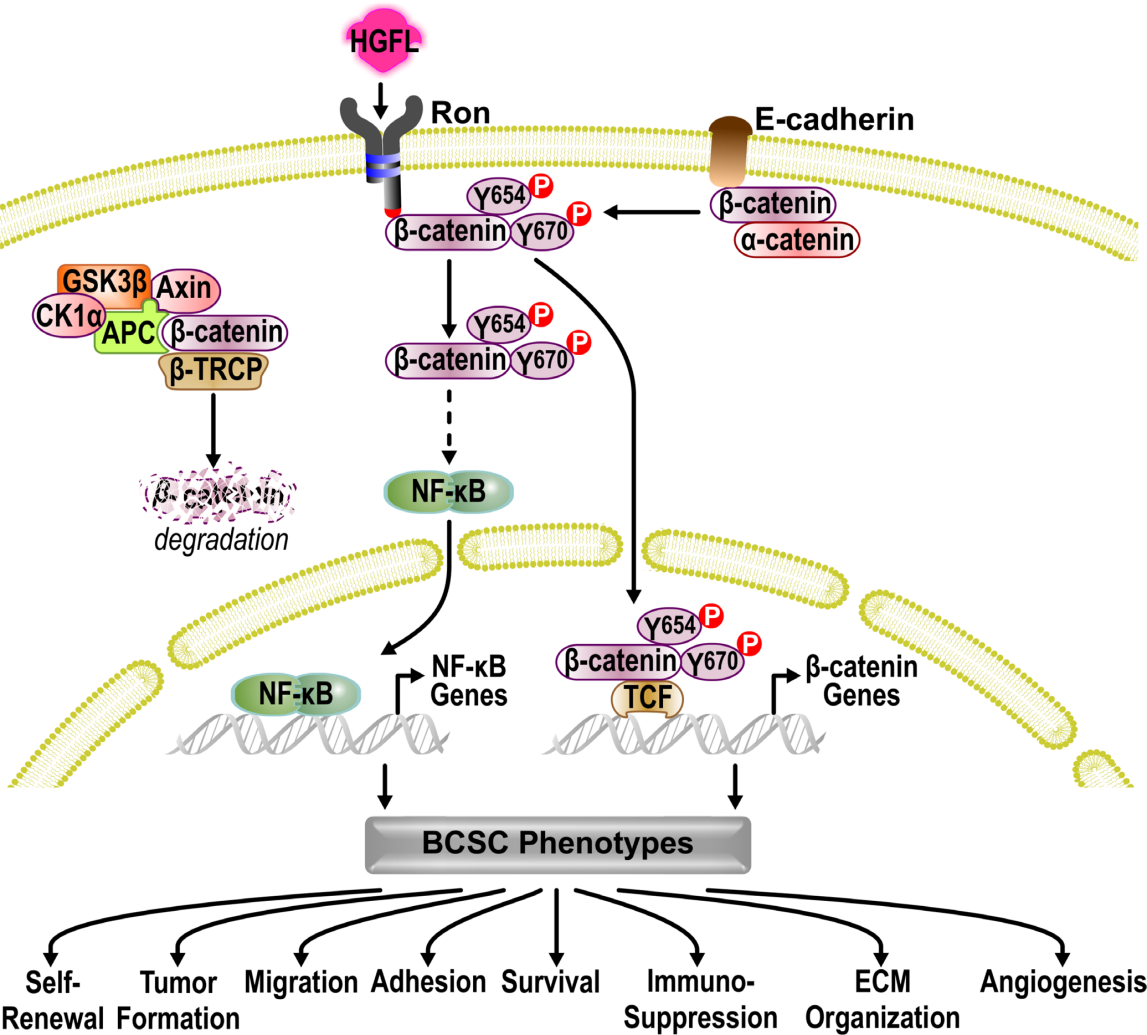
Mechanistic model In the absence of WNT ligands, β-CATENIN is recruited to the APC/AXIN/GSK3β/CK1α/β-TRCP destruction complex and is targeted for proteasome degradation, preventing canonical WNT/β-CATENIN signaling. Activation of HGFL-RON signaling within BCSCs bypasses the canonical β-CATENIN activation and supports the non-canonical activation of β-CATENIN. In the absence of HGFL-RON signaling, β-CATENIN is recruited by E-CADHERIN/*CDH1* to the cell membrane to function as an adaptor protein and transcriptional regulator. Activation of HGFL-RON signaling recruits and activates β-CATENIN via phosphorylation of β-CATENIN on tyrosine residues 654 (Y654) and 670 (Y670). This β-CATENIN activation then allows for the subsequent activation of NF-κB. Transcription of both NF-κB and β-CATENIN target genes then supports BCSC phenotypes by stimulating BCSC self-renewal, tumor formation, migration, adhesion, and survival as well as by altering the extracellular matrix (ECM) organization, angiogenesis, and anti-tumor immune responses, enhancing breast cancer growth.

We have previously shown that HGFL-RON signaling induces the non-canonical activation of β-CATENIN through tyrosine phosphorylation of β-CATENIN residues Tyrosine 654 and Tyrosine 670 [23]. To examine the involvement of the non-canonical activation of β-CATENIN downstream of RON in regulating BCSC phenotypes and the activation of NF-κB, we introduced a Flag-tagged wild-type (WT) β-CATENIN construct into β-catenin−/− cells. This WT β-CATENIN construct is capable of being activated by RON-mediated phosphorylation on tyrosine residues 654 and 670 or through canonical β-CATENIN signaling. In addition to the introduction of a WT β-CATENIN construct, a Flag-tagged double mutant (DM) β-CATENIN where tyrosine residues 654 and 670 were replaced with phenylalanine [23] was also utilized to reconstitute β-CATENIN expression. This mutant cannot be phosphorylated by RON but can undergo activation through canonical WNT signaling. Figure 5F demonstrates similar levels of Flag-tagged β-CATENIN expression in β-catenin−/− WT and β-catenin−/− DM reconstituted cells. However, we noticed that β-catenin−/− DM cells had a significant decrease in the phosphorylation of NF-κB compared to β-catenin−/− WT cells (Figure 5F), suggesting that RON-mediated tyrosine phosphorylation of β-CATENIN regulates the activation of NF-κB downstream of RON. Additionally, mammosphere formation analyses showed that expression of exogenous WT β-CATENIN rescued the self-renewal defect observed in β-catenin−/− cells, whereas expression of DM β-CATENIN had no effect in the self-renewal of β-catenin−/− cells (Figure 5G). These data demonstrate that HGFL-RON signaling promotes tumorigenesis by supporting BCSC phenotypes through the non-canonical activation of β-CATENIN and the subsequent activation of NF-κB rather than by canonical WNT/β-CATENIN signaling (Figure 6).

## DISCUSSION

Currently, first line of treatment against breast cancer involves the use of chemotherapy, radiotherapy, hormonal therapy, and/or targeted therapy to eradicate rapidly proliferating cells present within the tumor [1, 10]. However, a large portion of patients develop recurrence and therapeutic resistance following treatment [2–5], highlighting a lack of effectiveness of existing therapies for breast cancer patients. Mounting reports support the existence of a small subpopulation of breast cancer cells with tumor-initiating capabilities, named Breast Cancer Stem Cells (BCSCs), which possess stem cell properties and give rise to the non-tumorigenic and rapidly proliferating cells that comprise the bulk of the tumor [4, 10, 14]. These BCSCs are highly resistant to therapies currently available in the clinic and contribute to tumor relapse [3, 4, 8, 10, 13]. Recently, a strong effort has been made to focus in the development of new therapeutic strategies to effectively target and reduce BCSCs in patients [4, 31].

The RON receptor tyrosine kinase and its ligand, HGFL, play an important role in breast development, with HGFL-Ron signaling being required for normal mammary ductal morphogenesis and terminal end bud formation, processes which require pluripotent mammary stem cells [27]. RON and HGFL have also been strongly implicated in breast cancer progression, metastasis, and therapeutic resistance, being associated with poor prognosis in patients [6, 17, 18, 24–26]. However, it is not known whether RON signaling promotes breast tumor growth and progression through regulation of the BCSC subpopulation and to what extent its ligand HGFL influences this regulation.

Numerous reports have established the importance of receptor tyrosine kinases in regulating BCSC phenotypes, such as mammosphere formation, self-renewal, and tumor formation [12, 32–34]. Results reported herein are the first to reveal a critical role for HGFL-RON signaling in regulating BCSC functions and maintenance. Our studies demonstrate that depletion of HGFL or RON decreases the mammosphere formation, self-renewal, and numbers of both human and murine BCSCs, resulting in reduced tumor formation and growth as evidenced by our *in vivo* syngeneic transplantation experiments and the use of two clinically-relevant models of breast cancer [6, 17, 18]. Additionally, our data supports that both systemic HGFL and tumor-cell produced HGFL [6] regulate BCSCs. However, the extent to which systemic and tumor-cell produced HGFL regulate breast tumorigenesis remains to be determined. This information highlights the importance of ligand-dependent activation of RON during breast tumorigenesis and identifies two therapeutic strategies to target this signaling pathway in BCSCs, by either blocking HGFL binding to RON or inhibiting RON kinase activity.

Several monoclonal antibodies and receptor tyrosine kinase inhibitors with affinity for RON are currently in phase I/II clinical trials and have shown effectiveness in preclinical models of cancer [6, 28–30, 35, 36]. In this report, the therapeutic implication of inhibiting HGFL-RON signaling in BCSCs was further examined using two RON tyrosine kinase inhibitors, BMS-777607 and Foretinib. Data presented here shows that pharmacologic inhibition of RON reduces BCSC self-renewal, positing the HGFL-RON signaling as a novel therapeutic target to diminish BCSCs in patients. Based on this, we anticipate that inclusion of RON inhibitors as a monotherapy or in combination with other therapies targeting the rapidly-proliferating bulk cells will reduce tumor progression in breast cancer patients.

Regarding the mechanisms through which HGFL-RON signaling regulates BCSC phenotypes, our studies indicate that loss of HGFL-RON signaling in BCSCs is associated with a reduction in β-CATENIN and NF-κB signaling, two important BCSC regulators whose overexpression is associated with poor prognosis [6, 10, 20, 23, 31, 34, 37, 38]. The HGFL-RON signaling has been shown to stimulate β-CATENIN nuclear localization and transcriptional activity in breast cancers upon the phosphorylation of β-CATENIN on tyrosine residues 654 and 670 [6, 23, 26]. In addition, our previous reports have shown that RON regulation of NF-κB activation is both cell type and context dependent [39]. In innate immune cells, such as alveolar macrophages and tissue resident macrophages, RON signaling is associated with a decrease in the activation of NF-κB following lipopolysaccharide (LPS) challenge, limiting the macrophage-driven inflammatory response and decreasing tissue damage [21, 40–42]. However, in breast and prostate cancer epithelial cells and tumors, our laboratory has also shown that epithelial HGFL-RON signaling stimulates the activation of NF-κB by increasing the phosphorylation of IKKα/β and NF-κB while reducing IκBα accumulation, leading to increased survival and angiogenic chemokine production, stimulating tumor growth [6, 22, 39]. Interestingly, results described herein demonstrate that the HGFL-RON signaling-mediated β-CATENIN phosphorylation on tyrosine residues 654 and 670 is critical for increasing BCSC self-renewal and that this is partially accomplished through the downstream phosphorylation/activation of NF-κB after β-CATENIN activation. The cross-regulation between WNT/β-CATENIN and NF-κB has been extensively studied and is similar to the interactions reported between other self-renewal pathways, such as WNT/NOTCH, WNT/HEDGEHOG, and HEDGEHOG/NOTCH [38, 43]. Additionally, the interplay observed between β-CATENIN and NF-κB is consistent with a recent report showing that WNT/β-CATENIN signaling is required for NF-κB activation, which then regulates WNT/β-CATENIN activity for proper hair follicle development [43, 44]. Dissection of the mechanism of this interaction will provide novel insights on how these molecules regulate BCSC self-renewal upon RON activation and will support the use of RON inhibitors as a therapeutic strategy to target the cross-regulation between these two self-renewal pathways in breast cancer. Of note, our analyses suggest that in addition to NF-κB, other signaling molecules contribute to BCSC self-renewal downstream of β-CATENIN. Analysis of alternative pathways will strengthen the importance of the HGFL-RON-β-CATENIN axis in BCSC self-renewal.

Moreover, our transcriptional analyses revealed that activation of HGFL-RON signaling in BCSCs supports additional BCSC functions, such as proliferation, survival, and migration, which support tumor progression and metastasis [9, 11, 32]. Studies have shown that HGFL-RON signaling promotes several of these cellular functions [6, 16]; however, all these studies were performed using heterogeneous tumor samples containing mixed populations of BCSCs and bulk cells. Studies examining the role of HGFL-RON signaling in regulating these BCSC-specific activities and the identification of novel pathways required for these regulations will further define the mechanisms by which HGFL-RON signaling promotes breast cancer progression and support targeting this signaling pathway in late-stage breast cancers.

In addition to changes in cell-intrinsic mechanisms, HGFL-RON signaling in BCSCs also induced the transcription of genes that stimulate the metabolism of steroids as well as activities in the tumor microenvironment (TME), such as angiogenesis, extracellular matrix organization, and wound response, all of which promote tumor progression and recurrence [16, 21, 45]. Recent reports have established the importance of the TME in promoting cancer stem cell maintenance [33, 45, 46]. Publications from our laboratory have shown RON and HGFL expression on multiple cell-types within the TME, with activation of this signaling pathway leading to increased tumor burden associated with M2 anti-inflammatory macrophage polarization and decreased cytotoxic T-cell recruitment/activity, suppressing the immune system [6, 27, 47]. Data presented herein shows that HGFL-RON signaling in BCSCs supports an immunosuppressive TME, which might be regulated through inactivation of STAT4/JAK2 and Type-I IFN signaling. Analysis of the role of HGFL-RON signaling in influencing BCSC properties through modulation of the TME warrants further investigation. This is a critical aspect since inhibition of HGFL-RON signaling may prevent the communication between cells within the TME and BCSCs as well as reactivate the anti-tumor immune response, leading to more effective therapies for breast cancer patients.

Of importance, in addition to being activated by HGFL binding, RON can also be activated by homodimerization or heterodimerization with other receptor tyrosine kinases [21]. This alternative activation may lead to stimulation of genes that are activated in a HGFL-dependent and/or independent manner and thus may trigger different cellular activities depending on the mechanism of receptor activation. Nonetheless, the results herein show that similar cellular activities are affected in the absence of RON or HGFL and suggest that at least a subset of RON-mediated BCSC phenotypes are HGFL-dependent.

In conclusion, our results show that HGFL-RON signaling promotes mammary tumorigenesis by stimulating the BCSC self-renewal through a mechanism controlled by the RON-mediated tyrosine phosphorylation of β-CATENIN and the subsequent activation of NF-κB. In addition, our studies present novel BCSC functions and TME activities that might be regulated by this signaling pathway (Figure 6), which may explain the aggressive phenotype that is observed in RON overexpressing breast cancers. Overall, these data suggest RON and HGFL as novel therapeutic targets to suppress BCSC functions and effectively treat breast cancer patients, which could be extended to other cancers where RON is overexpressed.

## MATERIALS AND METHODS

### Mice

RON receptor tyrosine kinase (*TK*^+/+^), *PyMT TK*^+/+^, *PyMT TK*^−/−^, *MMTV-Ron Hgfl*^+/+^, and *MMTV-Ron Hgfl*^−/−^ female mice were generated and maintained in a FVB background as described [6, 17, 18]. *Hgfl*^−/−^ mice [27, 48] were crossed to *PyMT* to generate *PyMT Hgfl*^−/−^ mice. All mice were maintained under specific pathogen-free conditions and treated in accordance with protocols approved by the Institutional Animal Care and Use Committee of the University of Cincinnati.

### Cells and reagents

Human MCF-7 and T47D cells were obtained from ATCC and were recently authenticated (June/2016) using the PowerPlex16HS STR profiling (Genetica DNA Laboratories). R7 and β-cateninF/F cells were derived from mammary tumors from a transgenic *MMTV-Ron* mouse and from a transgenic *MMTV-Ron* mouse that was crossed into a homozygous β-catenin floxed background, respectively [18, 23]. Generation of MCF-7 PCI-Neo empty vector (EV), MCF-7 PCI-Neo *RON*, T47DshNT, T47Dsh*RON*, R7shNT, R7sh*Ron*, β-catenin−/−, β-catenin−/− WT, and β-catenin−/− DM cells has been previously described [19, 23]. R7sh*Hgfl* cells were generated after infecting R7 cells with *Hgfl* shRNA lentivirus (sequence: CCGGCGAGGTATGGTTGGGTACAATCT CGAGATTGTACCCAACCATACCTCGTTTTTG, Clone ID# NM_008243.2–793s1c1, Cincinnati Children's Hospital Medical Center) and selected with 1 μg/mL puromycin (Invitrogen, Cat# A1113803). R7 KD (3F7G10) cells were generated through CRISPR/CAS9 technology using guide RNA with homology to the RON kinase domain. The guide RNA sequence 5′-CACCGCTTACGACTCAGAGACTTGA-3′ and its reverse complement sequence 5′-AAACTCAAGTCTC TGAGTCGTAAGC-3′ were cloned into the pSpCas9 (BB)-2A-GFP (PX458) plasmid (Addgene, Cat# 48138). R7 cells were transfected with this plasmid and sorted based on GFP expression using the Aria Illu sorter (BD Biosciences). Single cell GFP+ clones were seeded in plates and stable cell lines containing RON targeting were confirmed through immunoblot and genomic analysis. For RON inhibition screenings, R7 cells were treated with increasing concentrations of BMS-777607 or Foretinib (Selleck Chemical, Cat# S1561 and S1111) for 24–72 hours.

### Cell growth assay

R7 and R7shNT cells were seeded at a density of 10,000 cells per well and MTT assays (Sigma, Cat# 298-93-1) were performed at 0, 24, 48, and 72 hours, as previously described [22].

### Mammary tumor cell isolation

Single-cell suspensions of mammary tumors were obtained by mechanical and enzymatic dissociation using 120 U/mL Collagenase I and 20 ug/mL DNase I (Worthington, Cat# LS004196 and LS002139) [6, 47].

### Flow cytometry analyses

Cells were stained with Aldefluor reagent as per manufacturer's instructions (Stemcell Technologies, Cat# 01700) and the percentage of cells with high Aldehyde Dehydrogenase (ALDH) activity were analyzed. Human breast cancer stem cell marker staining was determined by labeling human cells with CD44-APC and CD24-PE antibodies (BD Biosciences, Cat# 559942 and 555428). Dissociated mammary tumor cells and murine cell lines were stained with CD24-PE, CD29-FITC, CD31-APC, TER-119-APC (BD Biosciences, Cat# 553262, 561796, 561814, and 561033) and CD45-APC (Biolegend, Cat# 103111) antibodies, analyzed using the FACS Diva software, and sorted using the Aria Illu sorter (BD Biosciences) [47].

### Mammosphere (MS) formation assays

Cells were seeded at a density of 13,000 cells per mL in 6-well plates coated with 1% agarose and cultured as described [7, 15]. Cells were cultured under 3D-non-adherent conditions and the number of mammospheres greater than 50μm in diameter was counted after 14 days using an Axiovert S100TV microscope (AxioVision software-Carl Zeiss Microscopy) and ImageJ software (National Institutes of Health). Briefly, a 100 μm scale bar was used to calibrate the ImageJ software in order to measure the diameter of the mammospheres. Only mammospheres with a diameter greater than 50 μm were counted. The 50 μm threshold was established based on the average mammosphere size obtained for the control cells. Mammospheres were passaged using 0.05% trypsin (ThermoFisher Scientific, Cat# MT-25-051-CI) by culturing the same number of cells between the comparison groups under 3D-conditions. For HGFL stimulation experiments, R7sh*Hgfl* mammospheres were treated every 2 days with 100 ng/mL of recombinant HGFL (Cys672Ala, R&D Systems, Cat# 4306-MS/CF) and mammosphere formation ability was evaluated [19, 23]. For RON inhibition, R7 mammospheres were treated with 1 μM BMS-777607 or 0.01 μM Foretinib (Selleck Chemical, Cat# S1561 and S1111). For NF-κB inhibition, β-cateninF/F mammospheres were treated with 5 μM Bay 11-7085 (Enzo Life Sciences, Cat# EI279-0010). Mammosphere formation assays of primary BCSCs were performed by culturing the same number of cells between the respective comparison groups under 3D-conditions.

### Limiting dilution transplantation assays (LDA)

Lin^−^CD29^Hi^CD24^+^ sorted from R7, R7sh*Ron*, and R7sh*Hgfl* cells were orthotopically injected into the inguinal mammary fat pads of 9–12 week-old syngeneic FVB female mice following described protocols [14, 23, 49]. Tumor formation was examined 30 days post-injection. The number of tumors formed out of the number of sites injected was used to calculate the BCSC frequency and 95% confidence intervals for each group using the ELDA software [50].

### Immunoblot analyses

Cells were homogenized in RIPA buffer supplemented with protease inhibitors (Complete tablets, Roche Diagnostics, Cat# 11836153001), 1 mM AEBSF (ThermoFisher Scientific, Cat# 50-213-115), and 1 mM Na_3_VO_4_ (Sigma, Cat# S6508-10G). Antibodies for western analyses included: phospho-RON (R&D Systems, Cat# AF1947), RON-β (C-20) (Santa Cruz Biotechnology, Cat# SC-322), β-CATENIN (Cell Signaling Technology, Cat# 9582S and BD Biosciences, Cat# 610154), FLAG (Sigma, Cat# F1804), HGFL (T-19) (Santa Cruz Biotechnology, Cat# SC-6090), phospho-NF-κB p65 (Cell Signaling Technology, Cat# 3033S), NF-κB p65 (Cell Signaling Technology, Cat# 8242S), TUBULIN (Santa Cruz Biotechnology, Cat# SC-5286), and C4-ACTIN (Cincinnati Children's Hospital Medical Center). Peroxidase-conjugated secondary antibodies were applied and membranes were developed using Pierce ECL2 Western Blotting substrate (ThermoFisher Scientific, Cat# PI80196×3).

### RNA-Sequencing (RNA-Seq) analyses

RNA was isolated from R7 parental and R7, R7sh*Ron*, and R7sh*Hgfl* Lin^−^CD29^Hi^CD24^+^ BCSCs using the TRIZOL method (Invitrogen, Cat# 15596-018). RNA-Seq was performed on an Illumina HiSeq2500 for paired-end sequencing with 125 base pair reads. Data was analyzed using GeneSpring NGS software (Agilent Technologies) and sequences were aligned to the mm9 genome with annotations provided by UCSC. Aligned gene reads with a base quality threshold > = 30 were quantified and used to generate Reads Per Kilobase of transcript per Million mapped reads (RPKMs) for each transcript, which were log2-transformed, normalized using DESeq algorithm, and baselined to the median of all samples. Genes with > 3 reads in at least one sample were included in the analyses. Fold-changes were calculated as experimental group/control group to assess differential expression between the groups, with > 1, 2, or 5 thresholds used for differential regulation, depending on the analysis. Fold changes were used to identify the genes utilized in the heatmaps, which show normalized gene expression values, sample hierarchical clustering, and gene patterns. Functional enrichment analysis for differential genes was performed using ToppGene [51], which identifies gene ontology terms in the submitted gene list and provides an enrichment *P*-value using hypergeometric distribution with false discovery rate (FDR) correction. Data can be accessed at NCBI's Gene Expression Omnibus database (#GSE81941, https://www.ncbi.nlm.nih.gov/geo/query/acc.cgi?token=kredmgsijvklzcz&acc=GSE81941).

### Statistical analysis

Data are expressed as mean ± standard error of the mean (SEM). Statistical significance was determined by performing Student's *t*-test for pairwise comparisons or ANOVA for comparison of multiple groups using GraphPad Prism software. All *in vitro* experiments represent the average of at least triplicate experiments. LDA statistical analyses were performed using a pairwise chi-square test within the ELDA software [50]. Significance was set at *P* < 0.05.

## SUPPLEMENTARY MATERIALS FIGURES AND TABLES









## Abbreviations

HGFL: hepatocyte growth factor-like protein
TK: Tyrosine Kinase
BCSC: Breast Cancer Stem Cells
Lin^-^: Lineage negative
ALDH: Aldehyde Dehydrogenase
LDA: Limiting Dilution Transplantation Assays
ELDA: Extreme Limiting Dilution Analysis
TME: Tumor Microenvironment
ECM: Extracellular Matrix
MS: Mammospheres
WT: Wild-Type β-CATENIN
DM: Double Mutant β-CATENIN
NGS: Next Generation Sequencing
RPKMs: Reads per Kilobase of transcript per Million mapped reads
DESeq: Differential Expression analysis for Sequence count data
SEM: Standard Error of the Mean
LPS: Lipopolysaccharide
FDR: False Discovery Rate
