# Supplementary material for: HGFL-mediated RON signaling supports breast cancer stem cell phenotypes via activation of non-canonical β-catenin signaling
Source: Oncotarget. 2017 Jul 22;8(35):58918–33. doi: 10.18632/oncotarget.19441 (PMC5601703; doi:10.18632/oncotarget.19441)
Supplement: Supplementary file 1 [file oncotarget-08-58918-s001.pdf]

HGFL-mediated RON signaling supports breast cancer stem cell phenotypes via activation of non-canonical β-catenin signaling

SUPPLEMENTARY MATERIALS

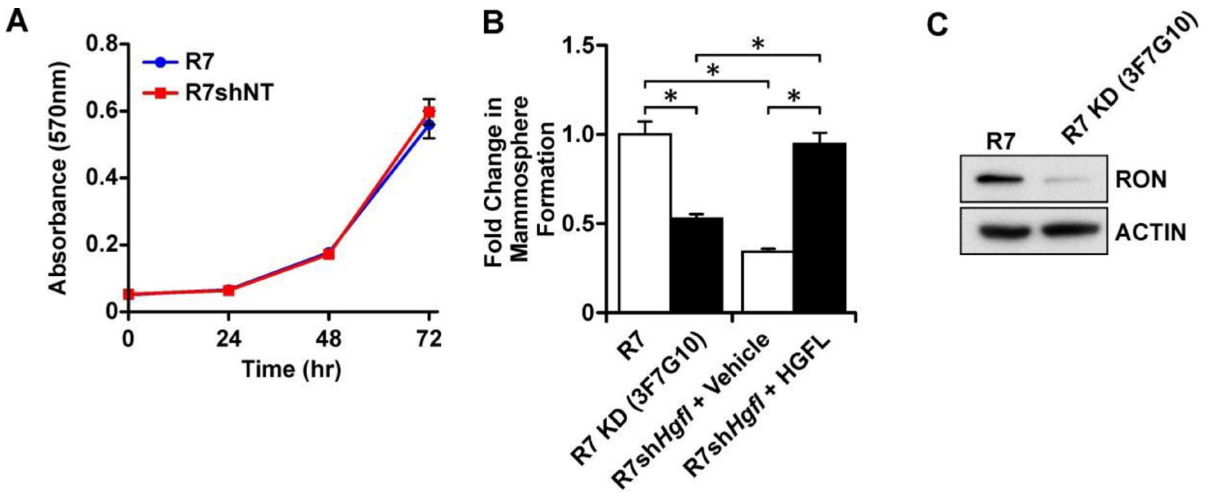

Supplementary Figure 1: R7 and R7shNT Cells Display Similar Phenotypes and Stimulation of RON Signaling Increases BCSC Mammosphere Formation and Self-renewal. (**A**) Cell viability of R7 cells, either untransduced or transduced with a non-targeting control shRNA (R7shNT), was measured at 0, 24, 48, and 72 hours using a MTT colorimetric assay ($n = 4$ independent experiments performed in triplicate). Bars represent average values ± SEM. (**B**) Quantification of the fold change in mammosphere formation obtained for R7 cells, the RON knockdown cell line R7 KD (3F7G10), and R7shHgfl cells either treated with vehicle or 100ng/ml of recombinant HGFL ($n = 2$ independent experiments performed in triplicate). Mammosphere formation in the control cells was set to 1. Bars represent average values ± SEM. *$P < 0.05$. (**C**) Western analysis for RON and ACTIN in R7 cells and R7 KD (3F7G10) cells.

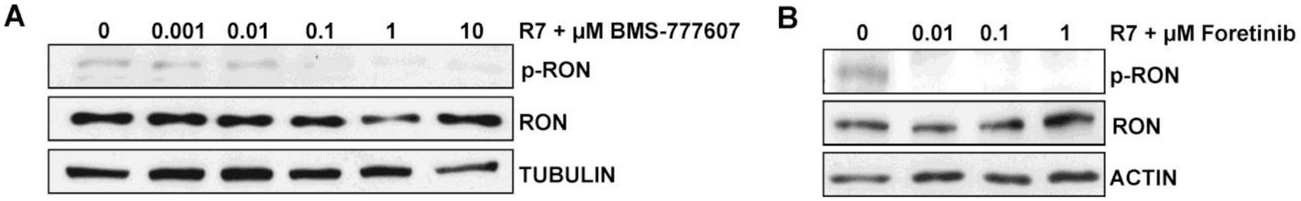

Supplementary Figure 2: BMS-777607 and Foretinib Inhibit RON Phosphorylation. (**A**) Immunoblot analysis for phosphorylated-RON, RON, and TUBULIN in R7 mammary tumor cells treated with increasing concentrations of BMS-777607 for 72 hours. (**B**) Western analysis for phosphorylated-RON, RON, and ACTIN in R7 cells treated with increasing concentrations of Foretinib for 24 hours.

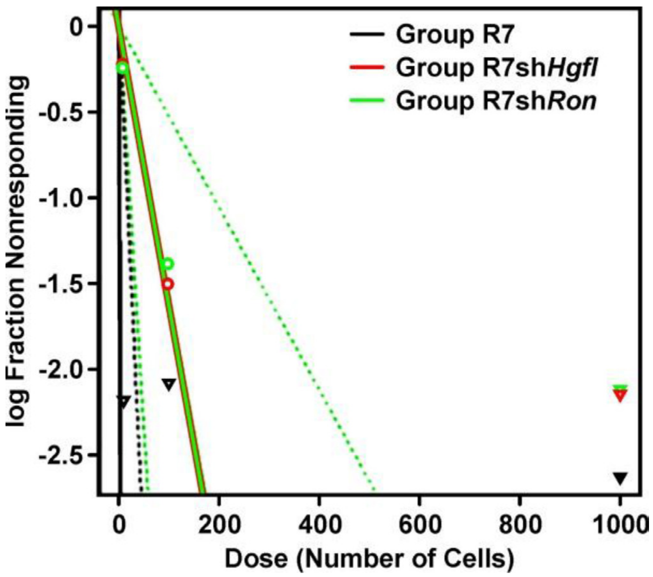

Supplementary Figure 3: Extreme-Limiting Dilution Analysis (ELDA) for R7, R7shRon, and R7shHgfl BCSCs. ELDA graph obtained for limited dilution transplantation assays performed using 10, 100, and 1000 Lin-CD29HiCD24+ BCSCs sorted from R7, R7shRon, and R7shHgfl cells. The number of tumors formed out of the number of sites injected was scored and graphed to determine the BCSC frequency (trend line) and the 95% confidence intervals (dotted lines) for each group.

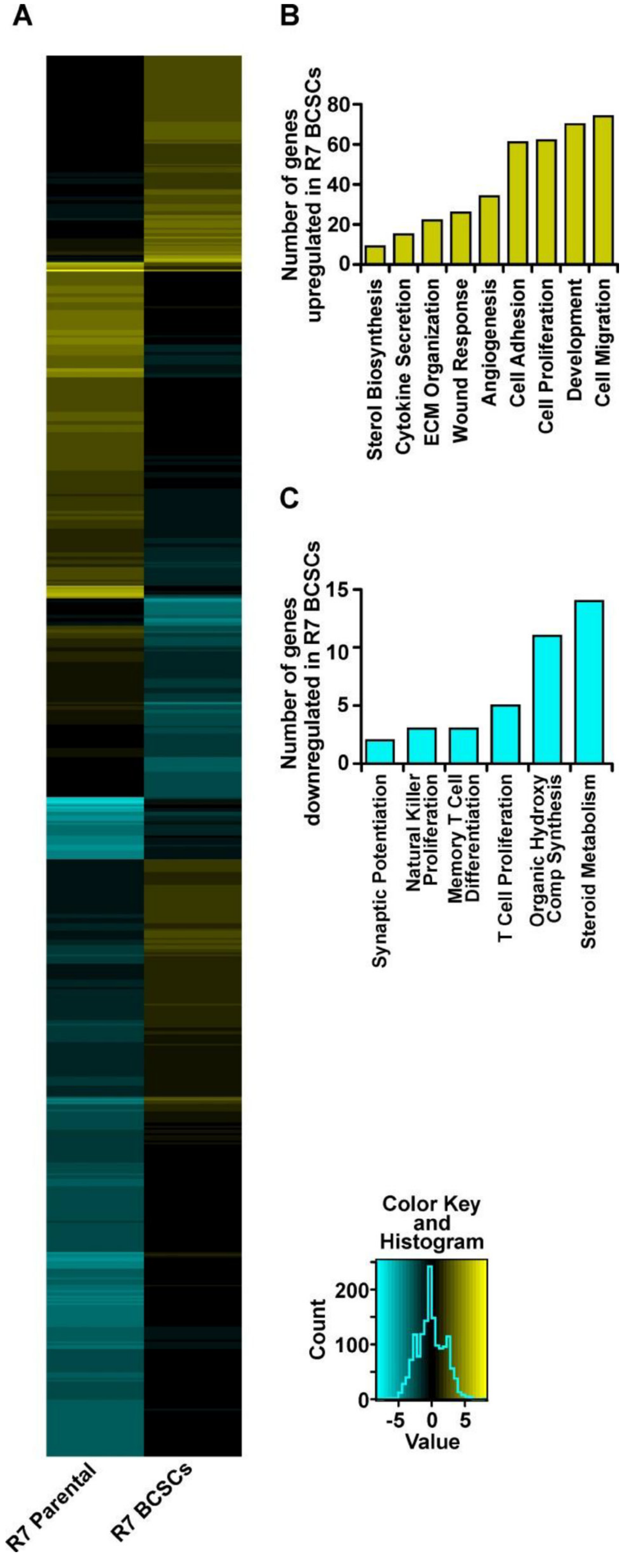

Supplementary Figure 4: HGFL-RON Signaling BCSCs Have an Aggressive Tumor-initiating Phenotype and Modulate the Tumor Microenvironment to Promote Breast Cancer Growth. (**A**) Gene expression profile for R7 Lin-CD29HiCD24+ BCSCs and R7 parental cells. Heatmap shows normalized gene expression values for genes with > 5-fold change in expression in R7 Lin-CD29HiCD24+ BCSCs compared to R7 parental cells. Histogram indicates the distribution of expression values. (**B**–**C**) Bar graphs depicting the top biological processes that are upregulated (B) and downregulated (C) in R7 Lin-CD29HiCD24+ BCSCs. See Supplementary Tables 1 and 2 for additional information.

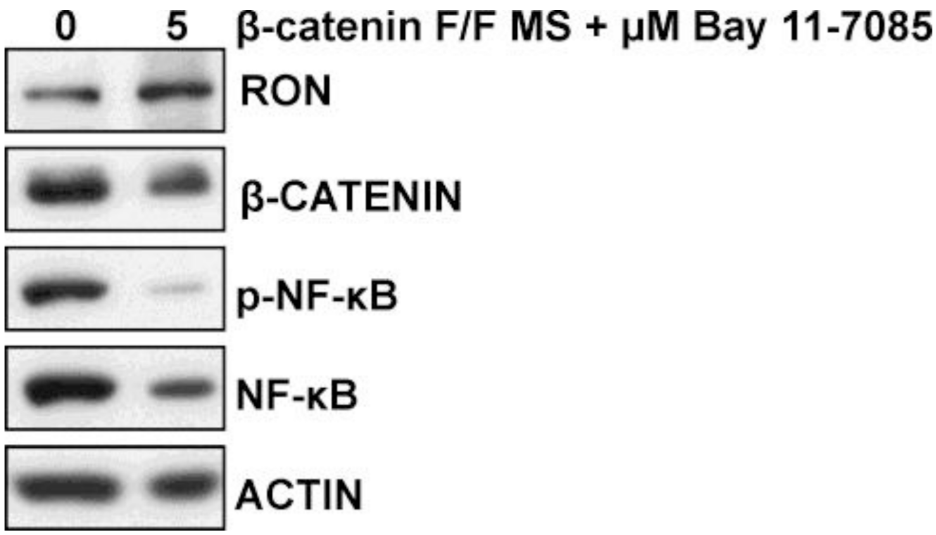

Supplementary Figure 5: Bay 11-7085 Inhibits NF-κB Phosphorylation Without Affecting RON and β-CATENIN Expression. Western blot images for RON, β-CATENIN, phosphorylated-NF-κB, NF-κB, and ACTIN expression in β-cateninF/F mammospheres treated with 0 and 5 µM Bay 11-7085 for 24 hours.

Supplementary Table 1: Top biological processes upregulated and downregulated in R7 Lin$^-$CD29^{Hi}CD24$^+$ BCSCs compared to R7 parental cells

Characteristic	Biological Process	Gene (*P*-value)
**Down in R7 BCSCs**	Steroid Metabolism	*Cyp2e1, Cyp27a1, Cyp27b1, Gfi1, Acox2, Kcnma1, Hsd17b1, Fgf15, Soat2, Vldlr, Stard5, Rdh9, Cyb5r2, Cyb5r1 (4.68E-06)*
	T Cell Proliferation	*Il12b, Cd244, Tnfrsf14, Ccr2, Il23a (1.47E-05)*
	Natural Killer Cell Proliferation	*Il12b, Cd244, Il23a (3.61E-05)*
	Memory T Cell Differentiation	*Il12b, Cd46, Il23a (3.61E-05)*
	Organic Hydroxy Compound Synthesis	*Cd244, Cyp27a1, Cyp27b1, Gch1, Chka, Gfi1, Acox2, Hsd17b1, Fgf15, Cyb5r2, Cyb5r1 (4.05E-05)*
	Synaptic Potentiation	*Ppp1r9a, Cx3cr1 (1.06E-04)*
	Cell Proliferation	*Nr4a1, F3, Serpinb5, Plau, Hes1, Ccl11, Cxcl12, Sfrp2, Angpt1, Htr2b, Rgcc, Id1, Nr1d1, Trp63, Il6, Sox9, Itgb3, Ptn, Scg2, Thbs1, Egr3, Atoh8, Has2, Hrh3, Id2, Igfbp3, Cdx2, Enpp2, Hmgcr, Hist1h2ac, Pim1, Agt, Cthrc1, Pla2g1b, Aldh3a1, Cx3cl1, Vipr1, Sgk1, Ifitm1, St8sia1, Aqp1, Csf1r, Smarca1, Tnk1, Cyp1b1, Spta1, Tnfrsf11a, Ace, Ptgir, Wisp2, Junb, Nfatc2, Dhcr24, Npy5r, Gper1, Vtcn1, Tmem119, Grpr, Sgk2, Egr4, Vsx2, Trib1 (3.59E-08)*
**Up in R7 BCSCs**	Cell Migration	*Enpp2, Mcam, F2rl1, F3, Agt, Plau, Ccl11, Cx3cl1, Hspb1, Cxcl12, Sele, Angpt1, Aqp1, Postn, Csf1r, Il6, Sox9, Cyp1b1, Itgb3, Srpx2, Gper1, Sema3d, Thbs1, P2ry6, Atoh8, Trpv4, Lgr6, Has2, Apcdd1, Cygb, Nr4a1, Scnn1g, Cthrc1, Pla2g1b, Hes1, Arrdc3, Mertk, Hspa1a, Hspa1b, Sfrp2, Fmod, Htr2b, Sgk1, Rgcc, Id1, Id2, Ifitm1, Shroom2, Igfbp3, Dnah11, Spta1, Dpysl5, Tnfrsf11a, Ace, Ptn, Nfatc2, Scg2, Dnah8, Krt16, Lhx4, Kif26b, Ldlr, Egr2, Egr3, Cd48, Trib1, Jph3, Acta2, Casq2, Serpinb5, Ccdc151, Lypd3, Kif1a, Dnah10 (9.06E-08)*
	Cell Adhesion	*Enpp2, Cdhr1, Mcam, Vit, F2rl1, Rs1, Agt, Sla2, C1qtnf1, Plau, Hes1, Ccl11, Mertk, Cx3cl1, Hspb1, Cxcl12, Sele, Chst10, Sfrp2, Angpt1, Abi3bp, Ppfia2, Rgcc, Id1, Egfl6, Cytip, Hapln1, Postn, Skap1, Cntnap2, Lypd3, Siglec1, Il6, Megf10, Sox9, Cyp1b1, Prss2, Spta1, Cldn15, Islr, Smoc2, Itgb3, Frem1, Ptn, Wisp2, Mmp24, Srpx2, Cacnb4, Kif26b, Vtcn1, Emilin1, Cldn2, Lgals4, Thbs1, Thbs2, Egr3, Cd36, Cd48, Trpv4, Has2, Cdh15 (1.30E-07)*
	Angiogenesis	*Cdx2, Enpp2, Acta2, Mcam, Nr4a1, F3, Agt, Plau, Hes1, Ccl11, Cx3cl1, Hspb1, Cxcl12, Sfrp2, Angpt1, Rgcc, Id1, Aqp1, Il6, Cyp1b1, Ace, Itgb3, Nsdhl, Ptn, Junb, Srpx2, Scg2, Gper1, Plxdc1, Thbs1, Thbs2, Egr3, Cd36, Has2 (3.25E-07)*
	Sterol Biosynthesis	*Cftr, Hmgcr, Hmgcs1, Fdft1, Insig1, Cyp51, Nsdhl, Dhcr24, Ch25h (1.49E-06)*
	Cytokine Secretion	*F2rl1, Agt, Cx3cl1, Angpt1, Htr2b, Rgcc, Nlrp1b, Postn, Csf1r, Srgn, Il6, Ssc5d, Vtcn1, Cd36, Trpv4 (1.77E-06)*
	ECM Organization	*Vit, Serpinb5, Fbn2, Agt, Col2a1, Sfrp2, Fmod, Abi3bp, Rgcc, Egfl6, Hapln1, Postn, Sox9, Cyp1b1, Prss2, Smoc2, Itgb3, Emilin1, Thbs1, Cd36, Lum, Has2 (2.74E-06)*
	Wound Response	*Cfh, Hmgcr, F2rl1, F3, Agt, C1qtnf1, Plau, Ccl11, Cx3cl1, Sele, Nlrp1b, Zfp36, Serpinc1, Il6, Tnfrsf11a, Ace, Cfb, Serping1, C4b, Npy5r, Gper1, Ldlr, Thbs1, Cd36, Otud7a, Trpv4 (3.20E-06)*
	Development	*Cdx2, Mns1, Enpp2, Mcam, Ccno, Atp8b1, Nr4a1, Rs1, F3, Fbn2, Agt, Cthrc1, Lmod1, Plau, Hes1, Ccl11, Cx3cl1, Ccdc151, Hspb1, Cxcl12, Col2a1, Sfrp2, Angpt1, Pla2g3, Rgcc, Id1, Aqp1, Trp63, Il6, Sox9, Cyp1b1, Ace, Itgb3, Ptn, Junb, Srpx2, Nfatc2, Scg2, Klhl6, Tbc1d30, Plxdc1, Kif26b, Dusp5, Casq2, Thbs1, Thbs2, Egr2, Egr3, Atoh8, Cd36, Hmgcr, Spdef, Islr2, Syt3, Sgk1, Fos, Id2, Ifitm1, Lrrn3, Trim67, Csf1r, Igfbp3, Brinp2, Nkx6-2, Mamstr, Gper1, Tmem119, Has2, Trib1, Cdh15 (5.56E-06)*

Supplementary Table 2: Fold Change in Gene Expression for R7 Lin$^-$CD29^{Hi}CD24$^+$ BCSCs Compared to R7 Parental Cells. See_Supplementary_Table 2

Supplementary Table 3: Effect of HGFL-RON signaling on the expression of genes shown to be upregulated in R7 $Lin^{-}CD29^{Hi}CD24^{+}$ BCSCs when compared to R7 parental cells

Cluster	Characteristic	Biological Process/ Pathway	Gene (*P*-value)
1	**Up in R7shHgfl BCSCs**	Inhibition of Cell Migration	*Ifitm1, Cyp1b1, Rgcc (3.49E-04)*
		Inhibition of Angiogenesis	*Rgcc, Cyp1b1, Hspb1 (4.22E-04)*
		Apoptosis	*Cyp1b1, Hspb1, Krt20 (4.22E-04)*
		Immune Response/Type I IFN Signaling	*Ifitm1, Hspb1, Hist1h3a*
		Inhibition of NF-κB Signaling	*Cyp1b1*
2	**Down in R7 BCSCs**	Immune Response	*Aicda, Gbp8, Itgb3, C4b*
		Type I IFN Signaling	*Mx2, Oas2, Zbp1*
		ECM Assembly	*Rhov, Fmod, Prelp*
		Sterol Biosynthesis	*Cyp51, Insig1*
		Apoptosis	*Csrnp3*
		Inhibition of WNT/β-CATENIN Signaling	*Dkk3*
		Inhibition of NF-κB Signaling	*Nr4a1*
3	**Up in R7shRon BCSCs**	Immune Response	*P2ry14, Aox3, Il2rb, Cxcl12, Cfh*
		ECM Assembly	*Synpo, Lum*
		Apoptosis	*Trp63*
		JAK/STAT Activation	*Jakmip2*
4	**Down in R7shRon BCSCs**	Inflammation/ Wound Healing	*Clec1a, Nfatc2, Skap1, Duox2, Krt16, Ifitm10*
		Cell Proliferation	*Egfl6, Vit, Id1, Ptn, Zmat4, Ppp1r26*
		Inhibition of Apoptosis	*Vit, Id1, Ptn, Zmat4*
		Angiogenesis	*Rem1, Egfl6, Id1, Smoc2*
		ECM Disassembly	*Col2a1, Stmn4, Prss2*
		Cell Migration	*Lypd3, Egfl6*
		NF-κB Signaling	*Il17re, Tnfrsf11a*
		Inhibition of Immune Response	*Serping1, Sla2*
5	**Up in R7 BCSCs**	ECM organization	*Mmp24, Fras1, Thbs2 (2.03E-02)*
		WNT/β-CATENIN Signaling	*Cdhr1, Acta2, Hapln1 (2.46E-02)*
		Cell Proliferation	*Cdx2, Vsx2, Egr4, Aldh3a1, Slc35g2, Tfec, Foxd4, Htr2b*
		Cell Adhesion	*Postn, Cldn2, Cdh15, Chst10, Cdhr1, Thbs2, Fras1*
		Inflammation/ Wound Healing	*Postn, Nlrp1b, Pla2g1b, F2rl1, Cdx2, Hp*
		Cell Migration	*Mmp24, Postn, Thbs2, P2ry6, Acta2, F2rl1*
		Stem Cell Maintenance	*Postn, Hapln1, Cdx2, Nkx6-2, Aldh3a1*
		Inhibition of Apoptosis	*Bex2, Igfbp3, Hspa1a, Htr2b, Tfec*
		NF-κB Signaling	*Pla2g1b, F2rl1, Hspa1a, Htr2b*
6	**Down in R7shHgfl BCSCs**	Stem Cell Maintenance	*Abi3bp, Srgn, Enpp2, Cd36, Kcnmb1, Lgr6, Has2 (4.30E-05)*
		ECM assembly	*Col24a1, Fbn2, Abi3bp, Srgn, Sparcl1 (2.27E-04)*
		Cell Proliferation	*St8sia1, Enpp2, Agt, Sfrp2, Ccl11, Scg2, Has2*
		Inflammation	*Agt, Sele, Cd36, Ccl11, Scg2*
		Angiogenesis	*Enpp2, Sfrp2, Ccl11, Scg2, Has2*
		Cell Migration	*Enpp2, Lgr6, Sele, Ccl11, Has2*
		Cell Adhesion	*St8sia1, Cd36, Ccl11, Has2*
		WNT/β-CATENIN Signaling	*Agt, Lgr6, Sfrp2*
		NF-κB Signaling	*Agt, Cd36*
		Inhibition of Immune Response	*Srgn, Rtn4rl1*

Supplementary Table 4: Effect of HGFL-RON Signaling on the expression of genes shown to be downregulated in R7 Lin$^-$CD29^{Hi}CD24$^+$ BCSCs when compared to R7 parental cells

Cluster	Characteristic	Biological Process/ Pathway	Gene (*P*-value)
1	**Up in R7 BCSCs**	FGFR Signaling	*Hhip, Fgf21, Esrp1 (7.54E-05)*
		Asparagine Catabolism	*Asrgl1 (7.52E-04)*
		Receptor Protein Tyrosine Kinase Signaling	*Hhip, Sh2d6, Fgf21, Esrp1 (1.47E-03)*
		Inflammation	*Ptx3, Esrp1 (1.50E-03)*
		Morphogenesis	*Esrp1, Hhip, Gbx1, Vldlr*
		Steroid Metabolism	*Stc2, Vldlr*
2	**Up in R7shRon BCSCs**	Immune Response	*Ccr2, Il12b, Il23a, Cyp27b1, Aqp9 (9.97E-07)*
		Apoptosis	*Ccr2, Il12b, Il23a (2.79E-05)*
		STAT4 Phosphorylation	*Il12b, Il23a (7.26E-06)*
		JAK2 Kinase Activation	*Il12b, Il23a (1.81E-05)*
		ECM Assembly	*Avil, Itih2, Ecm2*
		Differentiation	*Cyp27b1, Ptprt*
		Inhibition of β-CATENIN Signaling	*Ptprt*
3	**Down in R7 BCSCs**	Immune Response/ Type I IFN Signaling	*Lcp2, Ctss, Nlrp1a, Was, Tnfrsf14, Cd46, Entpd2, Kcnma1*
		Differentiation	*Mdk, Creb3l4, Dyrk4, Arl11, Gdap1l1, Kcnma1*
		Apoptosis	*G0s2, Arl11, Nlrp1a, Tnfrsf14, Atp2a1, Kcnma1*
		ECM Assembly	*Trim72, Tnnc1, Ablim2, Was, Cobl, Map6d1*
		Inhibition of NF-κB Signaling	*Gfi1*
4	**Up in R7shHgfl BCSCs**	Cell Adhesion	*Klra1, Klra4, Cd93, Opcml, Parvb, Azgp1*
		Immune Response/ Type I IFN Signaling	*Cd93, Cd74, Azgp1, Ncf2, Nuggc*
		Inhibition of Proliferation	*Nupr1, Azgp1, Sfn*
		Steroid Synthesis	*Soat2, Cyb5r2, Cyp2e1*
		Apoptosis	*Nupr1, Sfn*
		ECM Assembly	*Parvb, Ubxn11*

Supplementary Table 5: Fold Change in Gene Expression for R7shHgfl Lin-CD29HiCD24+ BCSCs and R7shRon Lin$^-$CD29^{Hi}CD24$^+$ BCSCs Compared to R7 Lin-CD29HiCD24+ BCSCs. See Supplementary_Table 5

Supplementary Table 6: Fold Change in Expression of Genes Related to β-CATENIN and NF-κB Pathways for R7 Lin$^-$CD29^{Hi}CD24$^+$ BCSCs and R7shRon Lin-CD29HiCD24+ BCSCs Compared to R7 Parental Cells. See Supplementary_Table 6
