# Supplementary material for: HGFL-mediated RON signaling supports breast cancer stem cell phenotypes via activation of non-canonical β-catenin signaling
Source: Oncotarget. 2017 Jul 22;8(35):58918–33. doi: 10.18632/oncotarget.19441 (PMC5601703; doi:10.18632/oncotarget.19441)
Supplement: Supplementary file 2 [file oncotarget-08-58918-s002.docx]

**Supplementary Table 2:** Fold Change in Gene Expression for R7 Lin^-^CD29^Hi^CD24^+^ BCSCs Compared to R7 Parental Cells.

| Gene Symbol | R7 BCSCs vs R7 Parental |
| --- | --- |
| Car6 | -304.437 |
| P2rx3 | -122.60697 |
| Cyp4f40 | -78.15086 |
| Cox6a2 | -58.438835 |
| Soat2 | -53.53437 |
| Gm6484 | -45.261444 |
| Slc19a3 | -43.062737 |
| G0s2 | -42.265278 |
| Fibin | -38.27798 |
| Cyb5r2 | -37.480526 |
| Dmrt1 | -37.480526 |
| 4930431P03Rik | -34.354496 |
| Abca12 | -34.290695 |
| Kcnma1 | -33.493233 |
| Serpinb9g | -31.89832 |
| Cdsn | -31.511126 |
| Aqp9 | -30.078808 |
| Dyrk4 | -28.908401 |
| B130024G19Rik | -28.708485 |
| Taf7l | -27.91103 |
| Slc1a1 | -27.869595 |
| Cobl | -26.316113 |
| Il23a | -23.923738 |
| Trem3 | -23.923738 |
| Avil | -22.802608 |
| P2rx3 | -22.717033 |
| 5430403N17Rik | -22.344263 |
| Klf15 | -22.344263 |
| Angptl6 | -22.15474 |
| Sspo | -20.894747 |
| Adm2 | -20.50612 |
| Grip1 | -19.93645 |
| Pabpc4l | -19.93645 |
| 1700013F07Rik | -19.13899 |
| Tmem125 | -18.341536 |
| Arl11 | -18.333752 |
| Cth | -18.001371 |
| Fgf21 | -17.692076 |
| 1700123M08Rik | -17.544077 |
| BB557941 | -17.544077 |
| Cyp27b1 | -17.544077 |
| 2810055G20Rik | -16.791422 |
| Gm7538 | -16.746616 |
| Cabp1 | -16.56111 |
| Slc7a3 | -15.612334 |
| Entpd2 | -15.18264 |
| 0610009L18Rik | -15.151702 |
| 1700001G11Rik | -15.151702 |
| Akr1b7 | -15.151702 |
| Pi16 | -15.151702 |
| 4930511M06Rik | -15.113422 |
| Chchd10 | -15.01076 |
| Esrp1 | -14.896176 |
| Slc6a12 | -14.466475 |
| Creb3l4 | -14.354244 |
| Ubxn11 | -14.354244 |
| Csn3 | -13.699896 |
| Rab39b | -13.670739 |
| Btg4 | -13.5567875 |
| Hsd17b1 | -13.5567875 |
| Map2 | -13.5567875 |
| BC048644 | -13.556785 |
| Cx3cr1 | -13.556785 |
| Gm15413 | -13.556785 |
| Gm17384 | -13.556785 |
| Kcnt2 | -13.174007 |
| Sh2d6 | -12.986407 |
| 2310034G01Rik | -12.759329 |
| Adamtsl3 | -12.759329 |
| Hyal6 | -12.759329 |
| Inhbe | -12.759329 |
| Vldlr | -12.714638 |
| Trim66 | -12.489868 |
| Pla2g2e | -12.217056 |
| Gm14327 | -12.031532 |
| 9530026P05Rik | -11.961869 |
| Fut1 | -11.961869 |
| Mei4 | -11.961869 |
| BC021614 | -11.758807 |
| Maats1 | -11.602974 |
| 3930402G23Rik | -11.164411 |
| 4930404I05Rik | -11.164411 |
| BC030499 | -11.164411 |
| Gm11454 | -11.164411 |
| Il12b | -11.164411 |
| Slc1a7 | -11.164411 |
| Tmem116 | -10.918448 |
| Trib3 | -10.5672035 |
| Cpne4 | -10.408988 |
| Armc2 | -10.366954 |
| Ptprt | -10.323227 |
| 9330179D12Rik | -10.312736 |
| Olfr1259 | -10.026271 |
| Fgf15 | -9.930781 |
| Acot2 | -9.865711 |
| Pbld2 | -9.739806 |
| Extl1 | -9.640649 |
| Adcy4 | -9.569495 |
| Calr4 | -9.569495 |
| Ctss | -9.569495 |
| E330034G19Rik | -9.569495 |
| Enkur | -9.569495 |
| Gm15663 | -9.569495 |
| Gm20778 | -9.569495 |
| Gm5486 | -9.569495 |
| LOC101055758 | -9.569495 |
| 4930483K19Rik | -9.50545 |
| Gadd45a | -9.3296795 |
| Ptx3 | -9.32079 |
| Tmem74 | -9.281463 |
| E230008N13Rik | -9.166876 |
| Gdap1l1 | -9.109584 |
| Hhipl1 | -9.088748 |
| Gbx1 | -9.023643 |
| Mef2b | -8.925704 |
| Zfp455 | -8.880409 |
| Cass4 | -8.798367 |
| Hhip | -8.784924 |
| Zfp811 | -8.772038 |
| 1700006H21Rik | -8.7720375 |
| Ankrd61 | -8.7720375 |
| Ccdc116 | -8.7720375 |
| Foxh1 | -8.7720375 |
| Palm2 | -8.7720375 |
| Trp53i11 | -8.7720375 |
| Wfdc12 | -8.7720375 |
| B4galnt2 | -8.623597 |
| H2afy3 | -8.5939455 |
| T | -8.5939455 |
| Itih2 | -8.307483 |
| Stbd1 | -8.253285 |
| E330023G01Rik | -8.021017 |
| 5430416N02Rik | -8.013653 |
| Zfp273 | -7.9895043 |
| Ablim2 | -7.97458 |
| Atp2a1 | -7.97458 |
| Cpsf4l | -7.97458 |
| D930028M14Rik | -7.97458 |
| Ecm2 | -7.97458 |
| Gfi1 | -7.97458 |
| Vsig1 | -7.97458 |
| Zfp750 | -7.97458 |
| Nmnat3 | -7.935459 |
| Dennd2d | -7.830041 |
| Slc6a9 | -7.814911 |
| Fbxl21 | -7.7345524 |
| Gm5072 | -7.7087617 |
| Chka | -7.624784 |
| Gm20767 | -7.575851 |
| 2210039B01Rik | -7.4480867 |
| 2810403D21Rik | -7.448086 |
| 1700109F18Rik | -7.445031 |
| Cd74 | -7.348418 |
| Gch1 | -7.298765 |
| Snhg12 | -7.2910786 |
| 1700017G19Rik | -7.177122 |
| 4930515G01Rik | -7.177122 |
| Ctrl | -7.177122 |
| Dcdc2a | -7.177122 |
| Mmp23 | -7.177122 |
| Pde5a | -7.177122 |
| Ppp1r9a | -7.177122 |
| Syt4 | -7.177122 |
| Trim72 | -7.177122 |
| Mir5133 | -7.120699 |
| Atp2a3 | -7.1043277 |
| Zfp329 | -7.0343046 |
| 2410006H16Rik | -6.929285 |
| Nupr1 | -6.8950253 |
| Nlrp1a | -6.875162 |
| Ccl6 | -6.8751564 |
| Gjc3 | -6.8751564 |
| Gm17396 | -6.8751564 |
| Oaz3 | -6.8751564 |
| Rdh9 | -6.8751564 |
| Serpine2 | -6.8751564 |
| Zfp422 | -6.8751564 |
| 1500012F01Rik | -6.8172183 |
| Rhbdd1 | -6.7927833 |
| E330033B04Rik | -6.7319245 |
| 1810010H24Rik | -6.5237603 |
| Cd93 | -6.493205 |
| Prss12 | -6.493205 |
| Gm867 | -6.4932027 |
| 1700120C14Rik | -6.4910517 |
| 2510049J12Rik | -6.3796644 |
| 5031426D15Rik | -6.3796644 |
| A530046M15Rik | -6.3796644 |
| AI197445 | -6.3796644 |
| Azgp1 | -6.3796644 |
| Calcb | -6.3796644 |
| Cd244 | -6.3796644 |
| Gm17597 | -6.3796644 |
| Ifna4 | -6.3796644 |
| Itgad | -6.3796644 |
| Lcp2 | -6.3796644 |
| Mansc1 | -6.3796644 |
| Mdk | -6.3796644 |
| Myf6 | -6.3796644 |
| Opcml | -6.3796644 |
| Parvb | -6.3796644 |
| Rab25 | -6.3796644 |
| Sfn | -6.3796644 |
| Slc6a4 | -6.3796644 |
| Stk39 | -6.3796644 |
| Tfap2a | -6.3796644 |
| Was | -6.3796644 |
| Dach2 | -6.3796635 |
| Iqcd | -6.3022275 |
| Snhg7os | -6.3022275 |
| Rab30 | -6.281764 |
| Gm14139 | -6.192274 |
| Sapcd1 | -6.1468053 |
| Aldh1l2 | -6.1389194 |
| Rhox2a | -6.1138444 |
| Cyp2e1 | -6.0917645 |
| Otub2 | -6.084842 |
| 1700101E01Rik | -6.040971 |
| Slc15a3 | -6.029445 |
| Tspyl4 | -5.9775667 |
| Als2cl | -5.973749 |
| A730056A06Rik | -5.958469 |
| Prss41 | -5.9279137 |
| Map6d1 | -5.9202733 |
| Gm5113 | -5.836722 |
| LOC100503002 | -5.8299036 |
| Cyb5r1 | -5.798416 |
| Stc2 | -5.788675 |
| Zfp599 | -5.78659 |
| Arntl2 | -5.762893 |
| Gpt2 | -5.7444777 |
| Gm16386 | -5.729299 |
| 1700022N22Rik | -5.7292986 |
| Ebf4 | -5.7292986 |
| 5730507C01Rik | -5.7292976 |
| Ncf2 | -5.7292976 |
| Tnnc1 | -5.7292976 |
| Rnase6 | -5.7289376 |
| Klra17 | -5.6742954 |
| 9330102E08Rik | -5.6656375 |
| 1700012D01Rik | -5.647754 |
| Dpp4 | -5.626864 |
| Resp18 | -5.5822062 |
| 4933417D19Rik | -5.5822053 |
| Aff2 | -5.5822053 |
| Ccr2 | -5.5822053 |
| Col25a1 | -5.5822053 |
| Cyp27a1 | -5.5822053 |
| D630008O14Rik | -5.5822053 |
| Dppa5a | -5.5822053 |
| Enpep | -5.5822053 |
| Gm10505 | -5.5822053 |
| Gm3948 | -5.5822053 |
| Gm8994 | -5.5822053 |
| Kif27 | -5.5822053 |
| Lef1 | -5.5822053 |
| Pde8a | -5.5822053 |
| Ppef2 | -5.5822053 |
| Serpinb9d | -5.5822053 |
| Tmem184a | -5.5822053 |
| 6030443J06Rik | -5.535877 |
| Klra1 | -5.493293 |
| Tnfrsf14 | -5.470333 |
| Wdr96 | -5.4444976 |
| 4632427E13Rik | -5.4428334 |
| 8430423G03Rik | -5.4428334 |
| Nuggc | -5.4428325 |
| 1700008O03Rik | -5.422714 |
| D130017N08Rik | -5.347345 |
| Gtpbp2 | -5.3371797 |
| Tmsb15b1 | -5.2942963 |
| Elfn2 | -5.263792 |
| Zfp712 | -5.2518563 |
| Spats1 | -5.251856 |
| Gm2366 | -5.2474756 |
| Zfp345 | -5.240399 |
| Klra4 | -5.230831 |
| B230206H07Rik | -5.185506 |
| 1810008I18Rik | -5.1563687 |
| 2310010J17Rik | -5.1563687 |
| Npm3-ps1 | -5.1563687 |
| Plxnc1 | -5.1563687 |
| Snora81 | -5.1563687 |
| Cd46 | -5.156368 |
| Veph1 | -5.156367 |
| 2610021A01Rik | -5.1525235 |
| Slc2a13 | -5.1086245 |
| Myom2 | -5.0990763 |
| Acox2 | -5.070429 |
| Asrgl1 | -5.06088 |
| Stard5 | -5.058902 |
| Sepw1 | -5.040123 |
| Zfp945 | -5.009677 |
| A4galt | 5.018067 |
| Gca | 5.018067 |
| Plxdc1 | 5.018067 |
| Gm16244 | 5.0193925 |
| Aqp1 | 5.0581393 |
| Emilin1 | 5.061113 |
| Fos | 5.065812 |
| Kctd12b | 5.0775704 |
| Cyp51 | 5.0852246 |
| Nrn1 | 5.09759 |
| Prrt2 | 5.1019816 |
| Gm13051 | 5.1036143 |
| Evc | 5.1222806 |
| Lpin1 | 5.128274 |
| Scd2 | 5.140637 |
| Acat3 | 5.151466 |
| Ces2e | 5.16197 |
| Celf6 | 5.163949 |
| Hist1h2bp | 5.1791463 |
| Serpina3h | 5.183481 |
| Ssc5d | 5.2085395 |
| 1700101I11Rik | 5.2362432 |
| A230083G16Rik | 5.2362432 |
| Aicda | 5.2362432 |
| Angpt1 | 5.2362432 |
| Col24a1 | 5.2362432 |
| Cpne7 | 5.2362432 |
| Dio2 | 5.2362432 |
| Dnah10 | 5.2362432 |
| F2rl3 | 5.2362432 |
| Gm12522 | 5.2362432 |
| Greb1 | 5.2362432 |
| Islr2 | 5.2362432 |
| Kcnj14 | 5.2362432 |
| Krt16 | 5.2362432 |
| Krt20 | 5.2362432 |
| Npy5r | 5.2362432 |
| Pnliprp2 | 5.2362432 |
| Ptcra | 5.2362432 |
| Rhov | 5.2362432 |
| Scd4 | 5.2362432 |
| Slc35g2 | 5.2362432 |
| Zfp286 | 5.2362432 |
| Zmat4 | 5.2362432 |
| P2ry6 | 5.236244 |
| Rapgef5 | 5.236244 |
| Serpinb9f | 5.236244 |
| Slc14a1 | 5.236244 |
| Cytip | 5.2482123 |
| Gm16796 | 5.2513885 |
| F2rl1 | 5.289135 |
| Hspb1 | 5.2937784 |
| Gm13279 | 5.310483 |
| Tagln | 5.327423 |
| C1qtnf1 | 5.3496394 |
| Acta2 | 5.354046 |
| 4930500J02Rik | 5.3828583 |
| Lrrn3 | 5.39025 |
| Hist1h1d | 5.394919 |
| Rtn4rl1 | 5.410786 |
| Frat2 | 5.42421 |
| Syt15 | 5.430179 |
| Gm15401 | 5.43018 |
| Cx3cl1 | 5.434586 |
| Rem1 | 5.4456944 |
| Cxcl12 | 5.500471 |
| Mlph | 5.5047693 |
| Gm13276 | 5.521852 |
| Gm10584 | 5.5347157 |
| Fdft1 | 5.5360126 |
| Krt28 | 5.567579 |
| 2410021H03Rik | 5.5675797 |
| 3110009F21Rik | 5.5675797 |
| 4930426L09Rik | 5.5675797 |
| 4930528D03Rik | 5.5675797 |
| 4933411G11Rik | 5.5675797 |
| 5430421F17Rik | 5.5675797 |
| Abcb9 | 5.5675797 |
| Abhd16b | 5.5675797 |
| Adam1b | 5.5675797 |
| Aldh3a1 | 5.5675797 |
| Aspn | 5.5675797 |
| Cd48 | 5.5675797 |
| Chst10 | 5.5675797 |
| Chst7 | 5.5675797 |
| Cldn2 | 5.5675797 |
| Clec1a | 5.5675797 |
| Clvs2 | 5.5675797 |
| Csrnp3 | 5.5675797 |
| Cstad | 5.5675797 |
| Cyp4f14 | 5.5675797 |
| D330023K18Rik | 5.5675797 |
| Dusp27 | 5.5675797 |
| Efcab12 | 5.5675797 |
| Egfl6 | 5.5675797 |
| Fam184b | 5.5675797 |
| Fam26d | 5.5675797 |
| Fam71a | 5.5675797 |
| Fam71e1 | 5.5675797 |
| Fbn2 | 5.5675797 |
| Fmo1 | 5.5675797 |
| Foxd4 | 5.5675797 |
| Gabra3 | 5.5675797 |
| Gbgt1 | 5.5675797 |
| Gbp8 | 5.5675797 |
| Gipc2 | 5.5675797 |
| Gjc2 | 5.5675797 |
| Glt1d1 | 5.5675797 |
| Gm10865 | 5.5675797 |
| Gm11128 | 5.5675797 |
| Gm14085 | 5.5675797 |
| Gm15638 | 5.5675797 |
| Gm1564 | 5.5675797 |
| Gm17281 | 5.5675797 |
| Gm17399 | 5.5675797 |
| Gm17689 | 5.5675797 |
| Gm5176 | 5.5675797 |
| Gm5615 | 5.5675797 |
| Gm5886 | 5.5675797 |
| Gria2 | 5.5675797 |
| Grpr | 5.5675797 |
| Hecw1 | 5.5675797 |
| Helt | 5.5675797 |
| Hrh3 | 5.5675797 |
| Igfbp3 | 5.5675797 |
| Il2rb | 5.5675797 |
| Ivl | 5.5675797 |
| Kcna2 | 5.5675797 |
| Kcnmb1 | 5.5675797 |
| Kctd14 | 5.5675797 |
| Lce3c | 5.5675797 |
| LOC100503338 | 5.5675797 |
| Lrrc46 | 5.5675797 |
| Lum | 5.5675797 |
| Mboat2 | 5.5675797 |
| Mc5r | 5.5675797 |
| Nptx1 | 5.5675797 |
| Otud7a | 5.5675797 |
| Oxct2a | 5.5675797 |
| Pde4c | 5.5675797 |
| Pla2g1b | 5.5675797 |
| Ppp1r26 | 5.5675797 |
| Ptgir | 5.5675797 |
| Rnf144a | 5.5675797 |
| Rnf32 | 5.5675797 |
| Rs1 | 5.5675797 |
| Scnn1g | 5.5675797 |
| Serpinc1 | 5.5675797 |
| Shroom2 | 5.5675797 |
| Slc2a4 | 5.5675797 |
| Smoc2 | 5.5675797 |
| St8sia1 | 5.5675797 |
| Stk32b | 5.5675797 |
| Syt9 | 5.5675797 |
| Tbc1d30 | 5.5675797 |
| Tfec | 5.5675797 |
| Timp4 | 5.5675797 |
| Tmem202 | 5.5675797 |
| Ucp1 | 5.5675797 |
| Upk1b | 5.5675797 |
| Vit | 5.5675797 |
| Vmn2r78 | 5.5675797 |
| Vsx2 | 5.5675797 |
| Wdr93 | 5.5675797 |
| Ybx2 | 5.5675797 |
| Cyp4a12a | 5.5675807 |
| Trim54 | 5.5675807 |
| Mrgprf | 5.57244 |
| P4ha3 | 5.6241136 |
| 1700003E16Rik | 5.6725965 |
| Fras1 | 5.6725965 |
| Gm4787 | 5.6725965 |
| P2ry14 | 5.6725974 |
| Hist1h2bk | 5.750787 |
| Kif1a | 5.765159 |
| Has2 | 5.796902 |
| E330013P04Rik | 5.818049 |
| Lypd3 | 5.818049 |
| Arrdc3 | 5.843138 |
| 9530027J09Rik | 5.856126 |
| Gm16432 | 5.8793635 |
| Hmgcr | 5.909855 |
| Ch25h | 5.93441 |
| AW011738 | 5.971156 |
| Cbr2 | 5.984279 |
| Tmem86a | 5.993043 |
| Junb | 6.0201836 |
| Lonrf1 | 6.031544 |
| Ptn | 6.0420012 |
| Gm11747 | 6.062012 |
| Mcam | 6.0853653 |
| Serpina3n | 6.090168 |
| March4 | 6.108951 |
| Best1 | 6.108951 |
| Cftr | 6.108951 |
| Cldn15 | 6.108951 |
| D6Ertd527e | 6.108951 |
| Hp | 6.108951 |
| Jakmip3 | 6.108951 |
| Jph3 | 6.108951 |
| Lrrc55 | 6.108951 |
| Msx3 | 6.108951 |
| Smarca1 | 6.108951 |
| Sprr2a3 | 6.108951 |
| Ucn2 | 6.108951 |
| Bhlhe40 | 6.198036 |
| Fam43a | 6.253136 |
| Etohd2 | 6.2579265 |
| LOC101056076 | 6.274662 |
| Mzf1 | 6.283494 |
| Dennd3 | 6.2998557 |
| Serpina3i | 6.3033066 |
| Frem1 | 6.327129 |
| Synpo | 6.366171 |
| Kif26b | 6.374557 |
| Cpxm1 | 6.3794894 |
| Mns1 | 6.3918233 |
| T2 | 6.3998523 |
| B230208H11Rik | 6.399854 |
| Ngb | 6.399854 |
| Enpp2 | 6.3998566 |
| 1810011O10Rik | 6.430223 |
| Col2a1 | 6.4446073 |
| Hmgcs1 | 6.473987 |
| 4931408D14Rik | 6.545304 |
| Ccno | 6.545304 |
| Gyltl1b | 6.5767217 |
| Sox9 | 6.581958 |
| Cthrc1 | 6.593789 |
| Ldlr | 6.598157 |
| Kcnk6 | 6.598519 |
| Pim1 | 6.6024175 |
| Casq2 | 6.6325765 |
| Dhcr24 | 6.7398167 |
| F3 | 6.7478766 |
| Atoh8 | 6.794649 |
| A330033J07Rik | 6.8704 |
| 1700109H08Rik | 6.875961 |
| Milr1 | 6.9539065 |
| 2010300C02Rik | 6.959474 |
| Agt | 6.959474 |
| Bex2 | 6.959474 |
| Clcnka | 6.959474 |
| Dgki | 6.959474 |
| Fam222a | 6.959474 |
| Gm20744 | 6.959474 |
| Hrc | 6.959474 |
| Map7d2 | 6.959474 |
| Slc14a2 | 6.959474 |
| Spdef | 6.959474 |
| Noxred1 | 6.9594755 |
| Arsi | 6.9816585 |
| Csf1r | 6.9816585 |
| Cys1 | 6.9816585 |
| Hs3st6 | 6.9816585 |
| Htr2b | 6.9816585 |
| Kcna4 | 6.9816585 |
| Lmod1 | 6.9816585 |
| Piwil2 | 6.9816585 |
| Pla2g3 | 6.9816585 |
| Scel | 6.9816585 |
| Serpinb5 | 6.9816585 |
| Sfrp2 | 6.9816585 |
| Srpx2 | 6.9816585 |
| Stmn4 | 6.9816585 |
| Vnn3 | 6.9816585 |
| Vtcn1 | 6.9816585 |
| Slc16a5 | 6.98166 |
| Srgn | 6.98166 |
| Trib1 | 6.990146 |
| Nsdhl | 7.0068703 |
| Cfb | 7.0561304 |
| Mamstr | 7.082894 |
| Dusp5 | 7.1105485 |
| Apcdd1 | 7.113162 |
| Rgcc | 7.1755924 |
| Egr2 | 7.4403696 |
| Gjb4 | 7.537018 |
| Prss27 | 7.5634627 |
| Trib2 | 7.587211 |
| Csrnp1 | 7.5972786 |
| Syt13 | 7.616354 |
| Slco4a1 | 7.692001 |
| Ppl | 7.7067885 |
| 1700026D08Rik | 7.745279 |
| Ifnz | 7.815066 |
| Il17re | 7.850287 |
| Tmem71 | 7.8543644 |
| Fam101a | 7.854366 |
| Msantd1 | 7.854366 |
| Thbs2 | 7.8548837 |
| Mxd3 | 7.8635206 |
| Nr1d1 | 7.9190626 |
| Il6 | 7.9465966 |
| Hspa1a | 7.963457 |
| Akr1c14 | 8.028908 |
| Glyat | 8.028908 |
| Scg2 | 8.028908 |
| 1700018L02Rik | 8.046131 |
| Zcwpw2 | 8.06381 |
| Lgals4 | 8.103711 |
| Itgb3 | 8.145269 |
| Thbs1 | 8.1504965 |
| Wisp2 | 8.181629 |
| Atp8b1 | 8.29072 |
| Rasd1 | 8.29072 |
| Spta1 | 8.29072 |
| 4930543E12Rik | 8.351369 |
| Amh | 8.351369 |
| Ccdc170 | 8.351369 |
| Cdh15 | 8.351369 |
| Cdhr1 | 8.351369 |
| Cutal | 8.351369 |
| Dpysl5 | 8.351369 |
| Duox2 | 8.351369 |
| Egr4 | 8.351369 |
| Fam167b | 8.351369 |
| Gm13718 | 8.351369 |
| Gm5434 | 8.351369 |
| Gm6297 | 8.351369 |
| Gpr88 | 8.351369 |
| Gprin3 | 8.351369 |
| Grid1 | 8.351369 |
| Hapln1 | 8.351369 |
| Hsf5 | 8.351369 |
| Ifitm10 | 8.351369 |
| Islr | 8.351369 |
| Lbx2 | 8.351369 |
| Nlrp1b | 8.351369 |
| Nwd1 | 8.351369 |
| Olfr1250 | 8.351369 |
| Paqr6 | 8.351369 |
| Serping1 | 8.351369 |
| Slc30a2 | 8.351369 |
| Fosb | 8.364767 |
| Cntnap2 | 8.37799 |
| Ggn | 8.412897 |
| Plin4 | 8.428646 |
| Sema3d | 8.4361725 |
| Dkk2 | 8.477726 |
| Siglec1 | 8.525356 |
| 5830403L16Rik | 8.538519 |
| Lce1g | 8.601956 |
| Cygb | 8.708622 |
| Dnah8 | 8.727073 |
| Fry | 8.727073 |
| Jakmip3 | 8.727073 |
| Syt3 | 8.727073 |
| Tnfrsf11a | 8.727073 |
| Trpv4 | 8.727073 |
| Cpa2 | 8.727075 |
| Egr3 | 8.752996 |
| Sv2c | 8.9452505 |
| Cyp4a12b | 9.076157 |
| Abi3bp | 9.094528 |
| Postn | 9.163429 |
| 5830444B04Rik | 9.283799 |
| Id2 | 9.298301 |
| Hist1h2ac | 9.579117 |
| BC037032 | 9.588664 |
| 1700007K13Rik | 9.599779 |
| Oas2 | 9.599781 |
| Far2 | 9.729456 |
| AI429214 | 9.743264 |
| Ccdc151 | 9.743264 |
| Cnksr1 | 9.743264 |
| Cyp1b1 | 9.743264 |
| Gm20594 | 9.743264 |
| Gm3764 | 9.743264 |
| Klhl32 | 9.743264 |
| Lhx4 | 9.743264 |
| Megf10 | 9.743264 |
| Npas4 | 9.743264 |
| Slc7a15 | 9.743264 |
| Zbp1 | 9.743264 |
| Ccdc64b | 9.890684 |
| Fbxl2 | 9.905633 |
| Sgk2 | 10.081438 |
| Dnah11 | 10.098968 |
| Prelp | 10.20688 |
| Fmod | 10.296042 |
| Galnt15 | 10.4724865 |
| Dkk3 | 10.756803 |
| Atp7b | 10.90884 |
| Hspa1b | 11.032332 |
| 2810029C07Rik | 11.1351595 |
| Aox3 | 11.1351595 |
| Arhgap36 | 11.1351595 |
| Fbxo27 | 11.1351595 |
| Jakmip2 | 11.1351595 |
| Myh1 | 11.1351595 |
| Pglyrp3 | 11.1351595 |
| Prss2 | 11.1351595 |
| Slc37a1 | 11.1351595 |
| Hist1h3a | 11.334 |
| Ace | 11.345193 |
| Ifitm1 | 11.345193 |
| Mx2 | 11.345193 |
| Plau | 11.562215 |
| Gna14 | 11.563373 |
| Sgk1 | 11.579443 |
| Apol8 | 11.603515 |
| Gm10825 | 11.636098 |
| 4930507D05Rik | 11.672092 |
| Sele | 11.724822 |
| A630033H20Rik | 12.217902 |
| Cd36 | 12.217902 |
| Ranbp3l | 12.358702 |
| Lgr6 | 12.527052 |
| Aff3 | 12.527054 |
| Ccl11 | 12.527054 |
| Klhl6 | 12.527054 |
| Wscd2 | 12.527054 |
| Gm11696 | 12.898576 |
| Tmem119 | 12.89904 |
| Insig1 | 12.973376 |
| Cdx2 | 13.090608 |
| 6430562O15Rik | 13.27393 |
| Nfatc2 | 13.381514 |
| Smtnl2 | 13.381514 |
| Id1 | 13.846066 |
| Rhbdl2 | 13.918946 |
| 6330403K07Rik | 13.918948 |
| Gper1 | 13.918948 |
| Slc25a2 | 13.918948 |
| Slc5a2 | 13.918948 |
| Tex19.1 | 13.918948 |
| A230028O05Rik | 13.963317 |
| Vipr1 | 13.963317 |
| Mertk | 14.626377 |
| Cfh | 14.83602 |
| Olig1 | 14.836027 |
| Cnnm1 | 15.310844 |
| Slc24a1 | 15.310844 |
| Gm16279 | 15.646935 |
| A230073K19Rik | 15.987746 |
| Cacnb4 | 16.290539 |
| Hes1 | 16.383093 |
| Nkpd1 | 16.702738 |
| Gm11149 | 17.247929 |
| Ppfia2 | 17.454145 |
| Sparcl1 | 17.454145 |
| Skap1 | 18.094631 |
| Trp63 | 18.11042 |
| Gm16740 | 18.22952 |
| Mgp | 19.199562 |
| Prom2 | 19.486523 |
| Brinp2 | 19.486528 |
| Gm10389 | 19.892178 |
| Ddit4l | 20.878422 |
| Fam189a1 | 20.878422 |
| Tprg | 20.878428 |
| Kprp | 21.52678 |
| Eif4ebp3 | 23.882498 |
| Fam180a | 24.435804 |
| Ranbp3l | 24.81359 |
| Xk | 25.054104 |
| Zfp36 | 25.899645 |
| Nr4a1 | 27.963154 |
| Nkx6-2 | 29.25431 |
| Trim67 | 38.055634 |
| C4b | 38.77819 |
| Phyhip | 38.973057 |
| Id3 | 44.551666 |
| Oacyl | 47.32443 |
| Mmp24 | 51.614788 |
| Lctl | 52.361427 |
| Gm10390 | 53.92821 |
| Gm21179 | 56.371742 |
| 8430408G22Rik | 71.866325 |
| Sla2 | 94.31784 |
| E130218I03Rik | 115.47274 |
| Tgm4 | 139.88542 |
| Tnk1 | 141.27731 |
