# Supplementary material for: HGFL-mediated RON signaling supports breast cancer stem cell phenotypes via activation of non-canonical β-catenin signaling
Source: Oncotarget. 2017 Jul 22;8(35):58918–33. doi: 10.18632/oncotarget.19441 (PMC5601703; doi:10.18632/oncotarget.19441)
Supplement: Supplementary file 3 [file oncotarget-08-58918-s003.docx]

**Supplementary Table 5:** Fold Change in Gene Expression for R7sh*Hgfl* Lin^-^CD29^Hi^CD24^+^ BCSCs and R7sh*Ron* Lin^-^CD29^Hi^CD24^+^ BCSCs Compared to R7 Lin^-^CD29^Hi^CD24^+^ BCSCs.

| Gene Symbol | Fold Change in Gene Expression | |
| --- | --- | --- |
|  | R7sh*Hgfl* BCSCs vs R7 BCSCs | R7sh*Ron* BCSCs vs R7 BCSCs |
| 0610009L18Rik | 20.77836 | 11.156172 |
| 1500012F01Rik | 2.0344288 | 1.1775119 |
| 1700001G11Rik | 7.333539 | 11.15617 |
| 1700008O03Rik | 6.111283 | 10.412427 |
| 1700012D01Rik | 9.641804 | 5.7748117 |
| 1700013F07Rik | 4.8890266 | 8.924936 |
| 1700018L02Rik | -1.9989347 | -3.5422957 |
| 1700022N22Rik | 3.5124974 | 7.4807577 |
| 1700026D08Rik | -1.2601961 | 2.5953548 |
| 1700101E01Rik | 4.3906217 | 2.938869 |
| 1700101I11Rik | -8.351369 | -3.7429366 |
| 1700109F18Rik | 8.488535 | 6.0601254 |
| 1700109H08Rik | -5.4937773 | -1.8490107 |
| 1700120C14Rik | 5.563019 | 7.4405036 |
| 1700123M08Rik | -1 | 5.9499583 |
| 1810010H24Rik | 3.2197886 | 2.3154724 |
| 1810011O10Rik | 1.8760815 | 2.187565 |
| 2010300C02Rik | -6.959474 | -2.3393354 |
| 2210039B01Rik | 2.6343722 | 1.6030192 |
| 2310010J17Rik | 2.1953108 | 2.404529 |
| 2310034G01Rik | 12.222566 | 9.668682 |
| 2410006H16Rik | 2.0963655 | 1.0533142 |
| 2410021H03Rik | -5.5675797 | -5.5675797 |
| 2510049J12Rik | 2.444513 | -1 |
| 2810055G20Rik | 1.9757794 | 4.4083033 |
| 2810403D21Rik | 5.533816 | 8.077078 |
| 3110009F21Rik | -2.2775824 | -1.4971749 |
| 3930402G23Rik | -1 | 2.2312343 |
| 4632427E13Rik | 2.1953104 | 4.9426427 |
| 4930404I05Rik | 4.8890266 | 5.9499583 |
| 4930426L09Rik | -5.5675797 | 1.8701891 |
| 4930431P03Rik | -1 | 4.462468 |
| 4930483K19Rik | -1.5615149 | 3.2543657 |
| 4930500J02Rik | 4.0046086 | 6.0128837 |
| 4930507D05Rik | -2.5146174 | -6.4002814 |
| 4930511M06Rik | 4.8890266 | 12.6436615 |
| 4930515G01Rik | 8.555797 | 2.9749792 |
| 4930528D03Rik | -4.5551643 | -3.7429368 |
| 4930543E12Rik | -3.4163735 | -1.8714683 |
| 4931408D14Rik | 3.922288 | 3.0279253 |
| 4933411G11Rik | -2.2775824 | -3.7429368 |
| 4933417D19Rik | 2.4640691 | 1.5916139 |
| 5031426D15Rik | -1 | 2.2312343 |
| 5430403N17Rik | 3.0734348 | -2.7837896 |
| 5430421F17Rik | -5.5675797 | -5.5675797 |
| 5730507C01Rik | -1.7081867 | 3.5622652 |
| 5830403L16Rik | -8.538519 | -8.538519 |
| 5830444B04Rik | -4.7156644 | -3.42979 |
| 6030443J06Rik | -1.4234889 | 2.351095 |
| 6330403K07Rik | -2.8469772 | -4.6786704 |
| 6430562O15Rik | -1.0700808 | -18.829687 |
| 8430408G22Rik | 3.6043499 | 2.9983037 |
| 8430423G03Rik | 5.2687454 | 1.6030192 |
| 9530026P05Rik | 4.8890266 | 15.047444 |
| A230073K19Rik | -2.4477096 | -1.0504762 |
| A230083G16Rik | 2.0489564 | 1.0686795 |
| A330033J07Rik | -6.8704 | -3.1157548 |
| A4galt | 3.245241 | 2.861645 |
| A530046M15Rik | 2.444513 | -1 |
| A630033H20Rik | -19.486528 | -1.6375346 |
| Abca12 | 2.444513 | -1 |
| Abcb9 | 2.1953104 | 1.7366045 |
| Abhd16b | -2.2775822 | -5.5675797 |
| Abi3bp | -2.50534 | 1.4572908 |
| Ablim2 | 24.445127 | 18.135471 |
| Ace | 3.7151408 | 6.1243563 |
| Acox2 | 3.7320273 | 5.370115 |
| Acta2 | -2.6852236 | -2.0419726 |
| Adam1b | -5.5675797 | -3.7429368 |
| Adcy4 | 25.667383 | 7.4374475 |
| Agt | -6.959474 | -1.8714684 |
| AI197445 | 3.6667695 | 4.462468 |
| AI429214 | -9.743264 | 5.5723996 |
| Aicda | 5.6274886 | 6.376456 |
| Akr1b7 | 2.444513 | 7.4374475 |
| Aldh3a1 | -4.5551653 | -5.5675797 |
| Amh | -1.3665493 | -8.351369 |
| Angpt1 | 1.8782102 | 2.73487 |
| Angptl6 | 2.4419448 | 1.542405 |
| Ankrd61 | 7.333539 | 7.4374475 |
| Aox3 | -1.3014755 | 3.254129 |
| Aqp9 | -1.1387911 | 2.1373591 |
| Arhgap36 | -11.1351595 | -11.1351595 |
| Arl11 | 41.271828 | 18.701893 |
| Armc2 | 2.444513 | 14.874895 |
| Asrgl1 | -8.351369 | -5.614404 |
| Atp2a1 | 2.444513 | 6.693703 |
| Atp7b | -4.7449627 | -1.949446 |
| Atp8b1 | -3.091005 | 1.012433 |
| Avil | 1.5806236 | 2.1390688 |
| AW011738 | 2.4317281 | 5.318735 |
| Azgp1 | 6.111283 | -1 |
| B130024G19Rik | 31.778671 | 46.85592 |
| B230206H07Rik | 4.7814684 | 2.2312343 |
| B4galnt2 | -9.128551 | 1.5998198 |
| BB557941 | 9.778053 | 11.156172 |
| BC021614 | 2.6343725 | -1.8714683 |
| BC030499 | -1 | 2.9749792 |
| BC037032 | -15.567118 | -2.9515219 |
| Best1 | 4.6415133 | 1.8320221 |
| Bex2 | -6.959474 | -6.959474 |
| C1qtnf1 | 2.483118 | 2.8969455 |
| C4b | 3.7304094 | 4.3497505 |
| Cabp1 | 3.0734348 | 3.4732091 |
| Calr4 | 11.000309 | 1.4874896 |
| Car6 | 1.7562487 | 3.7403796 |
| Casq2 | -1.3523148 | -3.9508784 |
| Cass4 | -6.959474 | -4.6786704 |
| Ccdc116 | 2.444513 | 2.9749792 |
| Ccdc151 | 1.0035703 | -2.1833801 |
| Ccdc170 | -6.832747 | -2.245762 |
| Ccl11 | -2.56228 | -1.122881 |
| Ccl6 | 3.512497 | 6.9464164 |
| Ccno | -1.8979849 | -5.614405 |
| Ccr2 | -1 | 2.9749792 |
| Cd36 | -2.6571794 | 1.442793 |
| Cd46 | 2.6343722 | 3.2060382 |
| Cd48 | -5.5675797 | -5.5675797 |
| Cd74 | 2.3599584 | -1.151673 |
| Cd93 | 1.7562484 | -2.8072026 |
| Cdh15 | -8.351369 | -8.351369 |
| Cdhr1 | -3.4163733 | -2.8072026 |
| Cdx2 | -8.540932 | -9.357341 |
| Ces2e | -6.8822594 | -13.006704 |
| Cfh | -1.0189182 | 8.486576 |
| Cftr | -9.743264 | -9.743264 |
| Ch25h | -3.2265751 | -3.1814966 |
| Chchd10 | 4.7418704 | 6.7326813 |
| Chst10 | -5.5675797 | -5.5675797 |
| Chst7 | -1.1387911 | -5.5675797 |
| Clcnka | 1.7562485 | -4.678671 |
| Cldn15 | 1.0035706 | -3.27507 |
| Cldn2 | -5.5675797 | -5.5675797 |
| Clec1a | 3.073434 | -2.4952912 |
| Cnksr1 | 2.7598186 | 1.8356855 |
| Cnnm1 | -2.0877838 | -1.2109501 |
| Cobl | 3.6667695 | 9.668682 |
| Col24a1 | -4.5551643 | 1.4249064 |
| Col25a1 | 26.88964 | 11.899917 |
| Col2a1 | -1.8220661 | -3.9056723 |
| Cox6a2 | -2.7837896 | 1.8701893 |
| Cpne4 | -2.7837896 | 5.343398 |
| Cpne7 | 1.3171864 | -5.614405 |
| Cpsf4l | 2.444513 | 2.9749792 |
| Cpxm1 | 2.6427815 | -1.520048 |
| Creb3l4 | 37.889954 | 22.312344 |
| Csn3 | 3.512497 | 1.8701893 |
| Csrnp3 | 2.4148412 | 1.7366041 |
| Cstad | -4.5551653 | -1.2476457 |
| Ctrl | -1 | 5.9499583 |
| Ctss | 6.111283 | 5.2062135 |
| Cutal | -3.4163733 | -1.4036013 |
| Cxcl12 | 1.3896705 | 6.21367 |
| Cyb5r2 | 2.444513 | -1 |
| Cyp1b1 | 2.5089257 | 1.1450138 |
| Cyp27b1 | -1 | 4.462468 |
| Cyp2e1 | 2.7180035 | 1.1450138 |
| Cyp4a12a | -5.5675807 | -5.5675807 |
| Cyp4a12b | -18.094631 | -18.094631 |
| Cyp4f14 | 2.8539035 | 1.7366045 |
| Cyp4f40 | 2.444513 | 1.4874896 |
| Cyp51 | 1.5754871 | 2.094098 |
| Cytip | -17.976952 | -59.08601 |
| D130017N08Rik | 1.6098943 | 2.3154724 |
| D630008O14Rik | 2.444513 | 6.693703 |
| D6Ertd527e | 3.0107117 | 1.7556881 |
| D930028M14Rik | 4.8890266 | 5.2062135 |
| Ddit4l | 2.7514555 | -1.7545015 |
| Dgki | 1.5806236 | 2.030491 |
| Dio2 | -4.911037 | -4.452804 |
| Dkk3 | 2.3295677 | 2.3292506 |
| Dnah10 | -6.832747 | -2.8072026 |
| Dnah11 | 1.101254 | -32.021183 |
| Dnah8 | -1.1387908 | -13.918948 |
| Dpysl5 | -1.1387911 | -8.351369 |
| Duox2 | -1.1387911 | -3.7429366 |
| Dusp27 | 6.3664002 | 5.209812 |
| Dyrk4 | 9.302628 | 8.490993 |
| E230008N13Rik | 2.6343725 | 1.6030194 |
| E330013P04Rik | -2.2775824 | -9.357344 |
| E330033B04Rik | 2.780727 | 2.6716988 |
| E330034G19Rik | 2.444513 | 2.2312343 |
| Ebf4 | 7.024995 | 9.425754 |
| Ecm2 | -1 | 8.924936 |
| Efcab12 | -2.2775824 | -3.7429368 |
| Egfl6 | -1.1387911 | -3.7429368 |
| Egr3 | 1.4675858 | 2.318286 |
| Egr4 | -8.351369 | -8.351369 |
| Eif4ebp3 | 2.1246862 | -23.425127 |
| Elfn2 | -18.220654 | -4.990583 |
| Enkur | 18.333843 | 16.362383 |
| Enpep | 2.444513 | 2.9749792 |
| Enpp2 | -2.1548228 | 1.1278025 |
| Entpd2 | 6.585932 | 2.4045289 |
| Esrp1 | -4.1756845 | -1.8714683 |
| Etohd2 | 1.7018964 | -4.2230635 |
| Extl1 | 2.0095537 | 1.829086 |
| F2rl1 | -2.3561199 | -1.8871951 |
| F2rl3 | 4.2442675 | 6.144908 |
| Fam167b | -1.3665495 | -5.6144056 |
| Fam180a | 4.5474286 | 3.6846545 |
| Fam184b | -1.5183882 | -3.7429366 |
| Fam222a | -5.693956 | -6.959474 |
| Fam26d | -2.2775824 | -2.4952912 |
| Fam71e1 | -5.5675797 | -1.2476456 |
| Far2 | 1.079634 | -3.2246034 |
| Fbn2 | -5.5675797 | -2.4952912 |
| Fbxl2 | 1.1453794 | -4.2438164 |
| Fbxl21 | 9.2203045 | 6.4120774 |
| Fbxo27 | -2.2775822 | -1.8714684 |
| Fgf15 | 1.1708324 | -2.8072026 |
| Fgf21 | -1.7793611 | -2.126669 |
| Fmod | 3.6486046 | 3.2344632 |
| Fos | 1.8559828 | 2.715047 |
| Foxd4 | -4.5551643 | -5.5675797 |
| Fras1 | -2.4673805 | -2.4329088 |
| Fut1 | 2.444513 | -1 |
| G0s2 | 17.11159 | 11.156172 |
| Gabra3 | -2.2775824 | -3.7429368 |
| Galnt15 | 2.4880185 | 2.8943403 |
| Gbp8 | 8.56171 | 7.6588693 |
| Gbx1 | -2.2775824 | -1.2476457 |
| Gca | -2.0147843 | -10.760943 |
| Gdap1l1 | 4.7418704 | 2.7785668 |
| Gfi1 | 8.555797 | 8.924936 |
| Ggn | -1.2915231 | 2.3280368 |
| Gipc2 | 3.732027 | 1.2022642 |
| Gjc2 | 2.6343722 | 3.606793 |
| Glt1d1 | -5.5675797 | -3.7429368 |
| Glyat | -32.013584 | -32.013584 |
| Gm10389 | -1.2407669 | -3.2582984 |
| Gm10390 | -3.924598 | -2.0216055 |
| Gm10505 | 7.333539 | 7.4374475 |
| Gm10584 | -2.4118903 | -1.7124802 |
| Gm10825 | 2.283123 | 2.7251332 |
| Gm10865 | -5.5675797 | -1.2441013 |
| Gm11128 | -5.5675797 | -1.3210366 |
| Gm11149 | 1.2315084 | -17.247929 |
| Gm11747 | 1.8888162 | -45.944237 |
| Gm13051 | -2.0877838 | -5.1036143 |
| Gm13279 | -1.1707165 | -2.0875058 |
| Gm14327 | 6.1468706 | 8.015097 |
| Gm15401 | 1.7709684 | -9.977102 |
| Gm15413 | 3.6667695 | 9.668682 |
| Gm15663 | -1 | 2.9749792 |
| Gm16279 | -1.1875614 | -5.3852196 |
| Gm16796 | -3.9276164 | -2.2559247 |
| Gm17396 | 4.3906207 | 4.8090577 |
| Gm17689 | -4.5551653 | -3.7429368 |
| Gm20594 | -2.6571796 | -6.550141 |
| Gm20744 | 1.4049988 | -2.3393354 |
| Gm20767 | 1.8333848 | 2.2312343 |
| Gm21179 | -56.371742 | -56.371742 |
| Gm2366 | 13.347042 | 9.675681 |
| Gm3764 | -9.743264 | -6.5501385 |
| Gm4787 | -2.4673805 | -12.164543 |
| Gm5072 | 4.4816074 | 10.412423 |
| Gm5113 | -1.2712088 | 2.7050953 |
| Gm5176 | -2.2775824 | -3.7429368 |
| Gm5434 | -8.351369 | -8.351369 |
| Gm5486 | -1 | 2.9749792 |
| Gm5615 | -2.2220318 | -1.4971749 |
| Gm6484 | 3.512497 | 1.0686796 |
| Gm7538 | 6.111283 | 1.4874896 |
| Gm867 | 2.6343727 | 1.8737514 |
| Gm8994 | 6.111283 | -1 |
| Gpr88 | -3.4163733 | -5.614405 |
| Gprin3 | -1.7081865 | 2.3154724 |
| Greb1 | 7.0964146 | 12.128802 |
| Gria2 | -5.5675797 | -3.7429368 |
| Grid1 | -8.351369 | -8.351369 |
| Grip1 | 2.444513 | 1.4874896 |
| Hapln1 | -8.351369 | -5.614405 |
| Has2 | -5.9993095 | -1.1326325 |
| Hecw1 | -5.5675797 | -3.7429366 |
| Helt | 1.0976552 | -5.5675797 |
| Hhip | -4.1756845 | -4.1756845 |
| Hhipl1 | -12.526704 | 1.4087138 |
| Hist1h1d | 2.6989403 | 2.9152944 |
| Hist1h3a | 2.4074802 | -1.1011474 |
| Hp | -9.743264 | -9.743264 |
| Hrc | 5.5933003 | 1.7098873 |
| Hrh3 | -2.2775824 | -1.8714684 |
| Hsf5 | -8.351369 | 1.0686795 |
| Hspa1a | -10.391472 | -10.978169 |
| Hspa1b | -4.768687 | -9.596046 |
| Hspb1 | 2.265307 | -1.0147839 |
| Htr2b | -4.5551653 | -2.9943497 |
| Id1 | -1.6191088 | -2.6644864 |
| Ifitm1 | 3.850237 | 1.3975041 |
| Ifitm10 | -3.416373 | -8.351369 |
| Ifnz | -2.1283267 | -2.0522008 |
| Igfbp3 | -5.5675797 | -5.5675797 |
| Il12b | -1 | 3.7187238 |
| Il17re | -3.2113907 | -7.850287 |
| Il23a | -1 | 4.462468 |
| Il2rb | 1.5367173 | 3.3396237 |
| Insig1 | 2.3286023 | 2.4271007 |
| Iqcd | 16.810814 | 11.755475 |
| Islr | -3.4163733 | -5.614405 |
| Itgb3 | 7.9658394 | 10.457793 |
| Itih2 | -2.7837896 | 2.1373591 |
| Ivl | -2.2775824 | -5.5675797 |
| Jakmip2 | 4.061324 | 11.354717 |
| Jakmip3 | 1.492811 | 2.3510947 |
| Jph3 | -3.985769 | -9.743264 |
| Kcna2 | -5.5675797 | 1.0686795 |
| Kcna4 | -2.2775822 | -1.4971749 |
| Kcnj14 | -1.7081865 | -2.8072026 |
| Kcnma1 | 2.444513 | 7.4374475 |
| Kcnmb1 | -5.5675797 | 1.0686795 |
| Kctd14 | -4.5551653 | -1.8714684 |
| Kif27 | 4.8890266 | 2.9749792 |
| Klf15 | -2.2775826 | -1.8714684 |
| Klhl32 | -1.3285897 | -4.3667603 |
| Klhl6 | -12.527054 | -8.421608 |
| Klra1 | 1.0444756 | -3.457741 |
| Klra4 | 1.3200415 | -3.4792898 |
| Kprp | -6.482348 | -1.7754956 |
| Krt16 | -1.1883036 | -11.22881 |
| Krt20 | 2.7807267 | -1.4971746 |
| Krt28 | -2.2775817 | -1.8714681 |
| Lce1g | -12.526704 | -2.7448204 |
| Lce3c | -5.5675797 | -4.9509745 |
| Lcp2 | 4.8890266 | 5.134814 |
| Lctl | -1.6550769 | -32.4215 |
| Lgr6 | -10.24912 | -1.4036013 |
| LOC100503002 | 2.7984889 | 2.6944094 |
| LOC100503338 | -5.5675797 | -5.5675797 |
| LOC101056076 | 4.1779065 | 4.125397 |
| Lonrf1 | -6.031544 | -6.031544 |
| Lrrc55 | -1.9928844 | -2.620056 |
| Lrrn3 | -2.097773 | -1.4239432 |
| Lum | -1.1387911 | 2.9388683 |
| Lypd3 | 1.2293737 | -13.918948 |
| Maats1 | -6.832747 | -4.491524 |
| Mamstr | -1.3203473 | -22.593243 |
| Map6d1 | 2.6343727 | 2.2264159 |
| Map7d2 | -1.4234887 | -6.959474 |
| Mboat2 | -5.5675797 | -5.5675797 |
| Mc5r | -5.5675797 | -5.5675797 |
| Mdk | 3.6667695 | 4.462468 |
| Mef2b | 5.738818 | 5.3878713 |
| Megf10 | 1.2544631 | 3.2060387 |
| Mgp | 10.118396 | 18.252764 |
| Mmp23 | 7.333539 | -1 |
| Mmp24 | -47.103806 | -20.373856 |
| Msx3 | -9.743264 | -6.55014 |
| Mx2 | 7.913385 | 11.427665 |
| Myf6 | 2.444513 | 1.4874896 |
| Myh1 | -2.2775822 | -11.1351595 |
| Ncf2 | 7.024995 | 2.6716988 |
| Nfatc2 | -1.7461463 | -5.3804717 |
| Ngb | 1.2772713 | -10.2930765 |
| Nkpd1 | 1.0976554 | -5.614405 |
| Nkx6-2 | -29.25431 | -29.25431 |
| Nlrp1a | 8.781244 | 6.94642 |
| Nlrp1b | -8.351369 | -8.351369 |
| Nmnat3 | 1.7562484 | -4.1756845 |
| Noxred1 | -6.9594755 | -3.1191146 |
| Npas4 | 3.136158 | 2.671699 |
| Npm3-ps1 | -1.1387911 | 2.1373591 |
| Nptx1 | -5.5675797 | -5.5675797 |
| Nr4a1 | 2.678092 | 3.0017364 |
| Nrn1 | -2.4291615 | 1.5978893 |
| Nuggc | 14.928109 | 5.4972878 |
| Nupr1 | 2.547205 | 1.6069471 |
| Nwd1 | 1.6098943 | 3.2060387 |
| Oacyl | -2.4199317 | -1.8179978 |
| Oas2 | 5.7477217 | 13.455646 |
| Oaz3 | 6.146869 | 5.8862877 |
| Olfr1259 | -2.7837896 | -2.7837896 |
| Olig1 | -6.453151 | -5.3024955 |
| Opcml | 2.444513 | 1.4874896 |
| Otud7a | -5.5675797 | -5.5675797 |
| Oxct2a | -5.5675797 | -3.7429368 |
| P2rx3 | 1.7836896 | 2.3210382 |
| P2rx3 | 2.6343722 | 8.015097 |
| P2ry14 | 1.7605716 | 5.231598 |
| P2ry6 | -4.7829223 | -4.9126043 |
| Palm2 | 4.8890266 | 3.7187238 |
| Paqr6 | -2.2775822 | -1.8714684 |
| Parvb | 3.6667695 | -1 |
| Pbld2 | 5.707807 | 4.2747188 |
| Pde4c | -4.5551653 | -5.5675797 |
| Pde8a | 2.444513 | -1 |
| Pglyrp3 | -11.1351595 | -11.1351595 |
| Phyhip | 1.7876097 | 3.0342867 |
| Pi16 | 19.556103 | 5.2062135 |
| Pla2g1b | -5.5675797 | -5.5675797 |
| Pla2g2e | 35.445442 | 15.618639 |
| Pla2g3 | 2.19531 | 4.742265 |
| Plin4 | 3.8498642 | 2.3908775 |
| Plxdc1 | 7.2931657 | 9.269638 |
| Pnliprp2 | -1.7081866 | -2.8072023 |
| Postn | -7.1235027 | -11.34457 |
| Ppef2 | 6.111284 | -1 |
| Ppp1r26 | -2.2775824 | -5.5675797 |
| Prelp | 2.5494978 | 3.6331124 |
| Prom2 | 1.1184797 | -2.381868 |
| Prss12 | 6.439577 | 1.0686795 |
| Prss2 | -1.5183883 | -3.7429368 |
| Prss41 | 5.299186 | 4.83898 |
| Ptcra | 1.3171864 | -5.614405 |
| Ptn | -1.1102588 | -2.2476196 |
| Ptprt | -1.1387911 | 2.0037742 |
| Ptx3 | -10.824044 | -8.142967 |
| Rab25 | 2.444513 | 5.2062135 |
| Ranbp3l | -1.2826979 | -4.497418 |
| Ranbp3l | -3.1213167 | -3.6980226 |
| Rapgef5 | -8.351369 | -1.8714687 |
| Rdh9 | 8.78124 | 7.213586 |
| Rem1 | 1.0319834 | -2.502421 |
| Resp18 | 2.444513 | 1.4874896 |
| Rgcc | 2.017313 | -1.0334976 |
| Rhbdl2 | -3.5587218 | -1.3367631 |
| Rhov | 3.5124974 | 4.5418887 |
| Rnase6 | 4.8890266 | 7.4374475 |
| Rnf32 | -1.5183883 | -5.5675797 |
| Rtn4rl1 | -2.0766194 | -1.2821113 |
| Sapcd1 | 4.267879 | -1 |
| Scd4 | -2.2775822 | 1.7069188 |
| Scel | 2.3050761 | 2.0705664 |
| Scg2 | -26.1922 | -3.5869815 |
| Scnn1g | -2.2775824 | -1.2476457 |
| Sele | -30.59932 | -3.8527715 |
| Sema3d | -1.5360442 | 6.4120774 |
| Sepw1 | 3.1129982 | -1.1944952 |
| Serpina3h | -20.14623 | -15.684765 |
| Serpina3i | -16.648767 | -14.479132 |
| Serpinb5 | 1.6464831 | -11.1351595 |
| Serpinb9d | 7.3335376 | 1.4874896 |
| Serpinb9f | -1.7081866 | -4.4915247 |
| Serpinb9g | 4.8890266 | 6.3218307 |
| Serpinc1 | -2.2775824 | -5.5675797 |
| Serping1 | -1.1387911 | -2.8072026 |
| Sfn | 6.111283 | 2.9749792 |
| Sfrp2 | -2.602951 | -1.6635276 |
| Sh2d6 | -4.1756845 | -1.8714683 |
| Shroom2 | 2.1953104 | 6.9464164 |
| Siglec1 | -8.525356 | -5.7313714 |
| Skap1 | 1.0132204 | -2.4329088 |
| Sla2 | 1.1701064 | -12.451943 |
| Slc14a2 | 2.809997 | 2.244227 |
| Slc15a3 | 11.493658 | 3.0957208 |
| Slc16a5 | -2.2775824 | -2.4952917 |
| Slc1a1 | 2.9891348 | 2.1373591 |
| Slc1a7 | 2.444513 | -1 |
| Slc24a1 | -1.0438919 | -2.0586154 |
| Slc25a2 | -2.2775822 | -1.7013348 |
| Slc2a13 | 3.6588514 | 3.9630203 |
| Slc2a4 | 6.805462 | 2.6716986 |
| Slc30a2 | -1.7081865 | -8.351369 |
| Slc35g2 | -3.4163733 | -2.8072026 |
| Slc6a4 | 8.555797 | 2.9749792 |
| Smarca1 | 1.630802 | 3.3812964 |
| Smoc2 | -2.2775824 | -5.5675797 |
| Snhg7os | 3.0734348 | -1.8714683 |
| Snora81 | 3.512497 | 5.8777385 |
| Soat2 | 1.4190487 | -4.678671 |
| Sparcl1 | -11.387912 | -2.3393354 |
| Spats1 | 4.39062 | 4.274718 |
| Sprr2a3 | 10.976259 | 3.3587065 |
| Srgn | -11.1351595 | -1.1516731 |
| Sspo | -1.5183883 | 2.137359 |
| St8sia1 | -5.5675797 | 1.0686797 |
| Stard5 | 1.2206295 | 2.1994402 |
| Stc2 | -2.1373453 | -1.7790895 |
| Stk32b | -5.5675797 | -5.5675797 |
| Stk39 | 2.444513 | 4.462468 |
| Stmn4 | -1.21148 | -11.1351595 |
| Synpo | 1.06566 | 2.209431 |
| Syt13 | 7.2902617 | 4.185661 |
| Syt15 | -5.5675797 | -3.7429368 |
| Syt9 | -5.5675797 | -5.5675797 |
| Tagln | -1.562972 | -3.5419562 |
| Tbc1d30 | 1.3171861 | -5.5675797 |
| Tex19.1 | -1.8979852 | -13.918948 |
| Tfec | -5.5675797 | -5.5675797 |
| Tgm4 | 1.0747194 | -71.2802 |
| Thbs2 | -3.6594017 | -4.0805397 |
| Tmem119 | 2.9250164 | 2.6258824 |
| Tmem125 | 2.444513 | 2.9749792 |
| Tmem184a | 18.333849 | 2.9749792 |
| Tmem202 | 2.1953104 | 3.2060387 |
| Tmem71 | 2.4880185 | 1.9592463 |
| Tmem74 | -2.8469775 | -1.6512954 |
| Tmem86a | 1.8022035 | 2.6297543 |
| Tmsb15b1 | 7.027395 | 1.7318482 |
| Tnfrsf11a | -2.8469775 | -4.1588187 |
| Tnfrsf14 | 4.8296824 | 7.2306867 |
| Tnk1 | -21.144844 | -141.27731 |
| Tnnc1 | 5.122392 | 3.4317513 |
| Trem3 | -1 | 2.9749792 |
| Trim54 | 10.757018 | -2.4952917 |
| Trim67 | -2.058043 | -2.1546211 |
| Trim72 | 7.333539 | 4.462468 |
| Trp53i11 | 2.444513 | 5.2062135 |
| Trp63 | 1.0578792 | 4.808441 |
| Ubxn11 | 16.241346 | 5.396612 |
| Ucp1 | -2.2775824 | -5.5675797 |
| Upk1b | 1.9757795 | -3.7429368 |
| Veph1 | -5.693955 | 2.244227 |
| Vit | 1.3171861 | -5.5675797 |
| Vldlr | -36.189262 | -36.189262 |
| Vmn2r78 | -1.5183883 | -5.5675797 |
| Vnn3 | -11.1351595 | -11.1351595 |
| Vsx2 | -5.5675797 | -5.5675797 |
| Vtcn1 | 2.5246067 | 1.8033965 |
| Was | 7.333539 | 20.82485 |
| Wdr96 | 10.960555 | 8.0248375 |
| Wfdc12 | 3.6667695 | 2.9749792 |
| Wscd2 | -12.527054 | -2.105402 |
| Xk | -5.124559 | 1.3358495 |
| Ybx2 | -5.5675797 | -1.8714684 |
| Zbp1 | 5.519637 | 4.732723 |
| Zcwpw2 | -1.2976924 | -34.101425 |
| Zfp273 | 3.1612468 | 2.5648308 |
| Zfp286 | 2.0489564 | 1.692076 |
| Zfp329 | -1.0511917 | 2.0334601 |
| Zfp455 | 7.683585 | 9.618115 |
| Zfp599 | 1.492811 | 2.671699 |
| Zfp712 | -3.4163735 | 2.671699 |
| Zfp811 | 24.445135 | 19.337364 |
| Zfp945 | 1.8330821 | 2.2273214 |
| Zmat4 | -1.7081865 | -8.351369 |
