# Supplementary material for: HGFL-mediated RON signaling supports breast cancer stem cell phenotypes via activation of non-canonical β-catenin signaling
Source: Oncotarget. 2017 Jul 22;8(35):58918–33. doi: 10.18632/oncotarget.19441 (PMC5601703; doi:10.18632/oncotarget.19441)
Supplement: Supplementary file 4 [file oncotarget-08-58918-s004.docx]

**Supplementary Table 6:** Fold Change in Expression of Genes Related to β-CATENIN and NF-κB Pathways for R7 Lin^-^CD29^Hi^CD24^+^ BCSCs and R7sh*Ron* Lin^-^CD29^Hi^CD24^+^ BCSCs Compared to R7 Parental Cells.

| Gene Symbol | Fold Change in Gene Expression | |
| --- | --- | --- |
|  | R7 BCSCs vs R7 Parental | R7sh*Ron* BCSCs vs R7 Parental |
| Akt1 | 1.4393508 | -1.3353282 |
| Akt2 | 1.1097614 | -1.1379285 |
| Akt3 | 1.0682386 | 1.6848584 |
| Ankrd6 | 1.0727026 | -1.3367938 |
| Apc | -1.2709188 | -1.4512157 |
| Atm | -1.1532313 | -1.3555104 |
| Axin1 | 1.0171909 | -1.0909309 |
| Axin2 | 1.0937933 | -1.6753433 |
| Bcl10 | 1.0399401 | -1.1479855 |
| Bcl2 | 1.1683786 | 1.0141871 |
| Bcl2l1 | -1.0125278 | -1.498084 |
| Birc2 | -1.0434837 | 1.2371686 |
| Birc3 | -1.7039075 | -1.3565654 |
| Blnk | 1.6424294 | -3.5634856 |
| Btk | -1.5949161 | -1.5949161 |
| Card11 | -1 | -1 |
| Cby1 | -1.0691605 | 1.2419695 |
| Ccl19 | -1 | -1 |
| Ccl2 | 1.249112 | -3.2500231 |
| Ccl4 | -1 | -1 |
| Cd14 | 1.1383138 | -1.4193416 |
| Cd40 | -1.4707674 | 1.3867817 |
| Cd40lg | -1 | -1 |
| Cer1 | -1 | -1 |
| Cflar | -1.98003 | -2.4184906 |
| Chuk | -1.5236883 | -1.8014497 |
| Csnk1a1 | -1.3415933 | -1.2125343 |
| Csnk2a1 | -1.4800317 | -1.7364279 |
| Csnk2a2 | -1.3346363 | -1.6561592 |
| Csnk2b | 1.2984719 | 1.0861236 |
| Ctnnb1 | 1.5007284 | 1.7586918 |
| Cxcl12 | 5.500471 | 34.178112 |
| Cxcl2 | -1.1104938 | -3.4354815 |
| Cxxc4 | 1.879677 | 4.71105 |
| Dact1 | 1.2451364 | 1.4851029 |
| Ddx58 | -1.0672222 | 2.8552017 |
| Dkk1 | -1 | -1 |
| Dkk2 | 8.477726 | 12.879375 |
| Dkk3 | 10.756803 | 25.055288 |
| Dkk4 | -1 | -1 |
| Dvl1 | -1.0924717 | -1.4134398 |
| Erc1 | -1.0313683 | -1.1125363 |
| Frat1 | 2.538785 | 3.1370766 |
| Fstl1 | 1.9807919 | 1.5446676 |
| Gadd45b | -4.1025934 | -4.545039 |
| Gsk3a | 1.0719041 | -1.1427778 |
| Gsk3b | -1.6924579 | -1.5871024 |
| Icam1 | 3.1635644 | 2.04016 |
| Ikbkb | -1.2060043 | -1.1734906 |
| Ikbkg | -1.1176275 | 1.2373518 |
| Il1b | -1 | 2.2312343 |
| Il1r1 | 1.0682986 | -1.0897439 |
| Irak1 | -1.1089914 | -1.4036195 |
| Irak4 | 1.0614474 | 1.6033098 |
| Lat | -1 | -1 |
| Lbp | 1.6333859 | 3.1140838 |
| Lck | -2.5065677 | -3.1273086 |
| Lrp1 | 1.2509704 | 1.1394377 |
| Lta | -1 | 10.412427 |
| Ltb | -1 | -1 |
| Ltbr | 1.1098677 | -1.0654403 |
| Ly96 | -1.0076219 | 2.790355 |
| Lyn | 1.254552 | 1.368171 |
| Malt1 | 1.168379 | 1.477961 |
| Map3k14 | -1.3272312 | 1.2678511 |
| Map3k7 | 1.2162479 | -1.0666707 |
| Mvp | 1.2574346 | 2.289693 |
| Myd88 | -1.3536139 | -1.1209155 |
| Nfkb1 | -1.3148423 | -2.0033875 |
| Nfkb2 | -1.4008749 | -1.8154639 |
| Nfkbia | 4.501002 | 1.475815 |
| Nkd1 | -1 | -1 |
| Nkd2 | -1.1458591 | -1.3566865 |
| Parp1 | 2.125078 | 1.6384046 |
| Pias4 | 1.3236057 | 1.4773759 |
| Pin1 | -1.0933119 | -1.783896 |
| Plau | 11.562215 | 13.063067 |
| Plcg1 | 1.128978 | 1.2097578 |
| Plcg2 | 1.6234719 | 1.7122147 |
| Prkcb | 1.7454146 | -1.4296266 |
| Prkcq | -1 | -1 |
| Psen1 | 1.1521553 | 1.0840249 |
| Ptgs2 | 1.5036955 | -1.0988626 |
| Ptpra | 1.5465281 | 1.7256352 |
| Rela | -1.0350064 | -1.1034055 |
| Relb | -2.1282778 | -3.2002773 |
| Ripk1 | 1.7766762 | 2.043466 |
| Rpsa | -1.1276236 | -1.1755271 |
| Senp2 | -1.1684948 | -1.5836042 |
| Sfrp1 | 1.6852282 | 1.0005382 |
| Syk | 2.198337 | 1.5195894 |
| Tab1 | -1.4833059 | -1.3877226 |
| Tab2 | -1.3924981 | -1.5146723 |
| Tab3 | -1.0321956 | 1.1446694 |
| Ticam1 | 1.6005665 | 2.7437546 |
| Ticam2 | 1.3918948 | 2.9749792 |
| Tirap | -1.7791195 | -1.6836293 |
| Tlr4 | 1.4181341 | 2.2017882 |
| Tnf | -1 | 6.693703 |
| Tnfaip3 | 1.6637052 | -2.184204 |
| Tnfrsf11a | 8.727073 | 2.0984502 |
| Tnfrsf13c | 1.950758 | 1.4123528 |
| Tnfrsf1a | 1.2451458 | 1.4858909 |
| Tnfsf11 | -1.594916 | -1.594916 |
| Tnfsf13b | 1.8418548 | -1.8276477 |
| Tnfsf14 | -1 | -1 |
| Tradd | -1.0934572 | 1.56136 |
| Traf1 | -3.4017706 | -6.366307 |
| Traf2 | -2.1082444 | -2.449532 |
| Traf3 | -1.0726597 | -2.1191583 |
| Traf5 | -1 | -1 |
| Traf5 | 1.2799709 | 3.4399242 |
| Traf6 | 1.0957668 | 1.2534131 |
| Trim25 | 1.3910602 | 1.8496397 |
| Tshb | -1 | -1 |
| Ube2i | -1.0877417 | -1.4014908 |
| Vcam1 | -1.1253427 | -3.7779546 |
| Wif1 | -1.7187893 | 3.419696 |
| Xiap | -1.127338 | 1.0557555 |
| Zap70 | -1 | 2.9749792 |
